# BAP1 deficiency causes loss of melanocytic cell identity in uveal melanoma

**DOI:** 10.1186/1471-2407-13-371

**Published:** 2013-08-05

**Authors:** Katie A Matatall, Olga A Agapova, Michael D Onken, Lori A Worley, Anne M Bowcock, J William Harbour

**Affiliations:** 1 Department of Ophthalmology & Visual Sciences, Washington University School of Medicine, St. Louis, Missouri, USA; 2 Department of Genetics, Washington University School of Medicine, St. Louis, Missouri, USA; 3 Bascom Palmer Eye Institute, University of Miami Miller School of Medicine, 900 S.W. 17th Street, Miami, FL, USA; 4 Sylvester Comprehensive Cancer Center, University of Miami Miller School of Medicine, Miami, FL, USA

**Keywords:** BAP1, Uveal melanoma, Differentiation, Stem cell, Metastasis, Tumor suppressor

## Abstract

**Background:**

Uveal melanoma is a highly aggressive cancer with a strong propensity for metastasis, yet little is known about the biological mechanisms underlying this metastatic potential. We recently showed that most metastasizing uveal melanomas, which exhibit a class 2 gene expression profile, contain inactivating mutations in the tumor suppressor *BAP1*. The aim of this study was to investigate the role of BAP1 in uveal melanoma progression.

**Methods:**

Uveal melanoma cells were studied following RNAi-mediated depletion of BAP1 using proliferation, BrdU incorporation, flow cytometry, migration, invasion, differentiation and clonogenic assays, as well as in vivo tumorigenicity experiments in NOD-SCID-Gamma mice.

**Results:**

Depletion of BAP1 in uveal melanoma cells resulted in a loss of differentiation and gain of stem-like properties, including expression of stem cell markers, increased capacity for self-replication, and enhanced ability to grow in stem cell conditions. BAP1 depletion did not result in increased proliferation, migration, invasion or tumorigenicity.

**Conclusions:**

BAP1 appears to function in the uveal melanocyte lineage primarily as a regulator of differentiation, with cells deficient for BAP1 exhibiting stem-like qualities. It will be important to elucidate how this effect of BAP1 loss promotes metastasis and how to reverse this effect therapeutically.

## Background

Uveal melanoma is a highly aggressive cancer that arises from melanocytes within the uveal tract of the eye. Uveal melanomas can be classified according to their transcriptomic signature into two prognostically significant subtypes. Class 1 uveal melanomas are less aggressive and rarely metastasize, whereas class 2 uveal melanomas are highly aggressive and commonly give rise to fatal metastatic disease [1,2]. We recently showed that inactivating mutations in the tumor suppressor *BAP1* occur almost exclusively in class 2 tumors and are strongly associated with metastasis, suggesting that BAP1 may function as a metastasis suppressor in uveal melanoma [3]. One patient in this report carried a germline *BAP1* mutation, indicating that *BAP1* mutations can give rise to a familial cancer syndrome. Since this report, somatic and germline *BAP1* mutations have been identified in a variety of other tumors, including mesothelioma, cutaneous melanoma, atypical cutaneous melanocytic tumors, lung adenocarcinoma, meningioma and renal cell carcinoma [4-9].

BAP1 (BRCA1-associated protein-1) is an ubiquitin carboxy-terminal hydrolase that was identified in a screen for proteins that interact with BRCA1 [10]. It was initially found to be mutated in a few breast and lung cancer cell lines, where it exhibited tumor suppressor activity upon re-introduction [10]. BAP1 has been suggested to function in several pathways, including DNA damage repair, cell proliferation and development [11-14].

In *Drosophila* the BAP1 homolog Calypso is a component of the PR-DUB Polycomb repressive complex, and its loss results in a developmental phenotype characterized by deregulated HOX gene expression [14]. This study showed that both Calypso and human BAP1 catalyze the removal of monoubiquitin moieties from histone H2A when in the presence of Asx or ASXL1, respectively. This activity of BAP1 opposes the H2A ubiquitinating activity of the PRC1 complex, which contains BMI1. Interestingly BMI1 is an oncogene involved in stem cell maintenance, and its over-expression leads to a loss of cell identity in multiple cancers [15]. We recently showed that BAP1 loss causes increased histone H2A ubiquitination in melanoma cells and melanocytes, and this hyperubiquitination was reversed by treatment with HDAC inhibitors, which inhibit BMI1 [16].

Another recent study found that BAP1 loss leads to a myelodysplastic syndrome (MDS) in mouse [17]. They found that the predominant BAP1-interacting proteins in the hematopoietic lineage are HCF-1, OGT, ASXL1/2, and FOXK1/2, which is consistent with other studies [12-14,18]. In contrast to the findings in *Drosophila*, however, BAP1 loss in mouse did not effect HOX gene expression, suggesting that BAP1 may have divergent roles across species.

Despite the recently renewed interest in BAP1, the precise cellular impact of BAP1 loss during tumorigenesis remains unclear. In this study, we wished to determine the function of BAP1 in uveal melanoma, where BAP1 loss appears to play a specific role in tumor progression and acquisition of metastatic capacity. Our findings suggest that a major role for BAP1 in this setting is to regulate transcriptional programs involved in maintaining a differentiated melanocytic phenotype and that loss of BAP1 triggers a loss of cell identity characterized by a primitive, stem-like phenotype.

## Methods

### Tissue culture

Three uveal melanoma cell lines OCM1A, 92.1, and Mel290 (kindly provided by Drs. J. Kan-Mitchell, M. Jager and B. Ksander, respectively) were used in this study. All three cell lines are wildtype for BAP1. 92.1 cells contain a GNAQ^Q209L^ mutation, OCM1A cells contain a BRAF^V600E^ mutation, and Mel290 cell lines are wildtype for both GNAQ and BRAF; all of the cell lines are wildtype for GNA11 [19]. These cell lines are well-established tools in the field of uveal melanoma research and their mutational status is representative of the spectrum seen in uveal melanoma. Due to the low frequency of BRAF mutations in uveal melanoma, OCM1A cells may not be representative of the vast majority of primary uveal melanomas. All uveal melanoma cell lines were grown in RPMI-1640 supplemented with 10% FBS, L-glutamine, and antibiotics at 5% CO_2._ Primary uveal melanoma samples were collected at the time of enucleation and informed consent was obtained for each patient. All samples were confirmed to be uveal melanomas by pathologic evaluation and melanoma cells were isolated and grown as previously described [20]. Primary uveal melanoma cells were grown on collagen-covered tissue culture plates in 5% CO_2_ and 4% O_2_ in MDMF medium which consists of HAM’s F12 (Lonza, Walkersville MD, USA) supplemented with 1 mg/ml BSA (Sigma-Aldrich, St Louis MO, USA), 2 mM L-glutamine (Lonza), 1X SITE (Sigma-Aldrich), 1x B27 (Gibco, Carlsbad CA, USA), 20 ng/ml bFGF (PeproTech Inc, Rocky Hill NJ, USA), 50 μg/ml Gentamicin (Sigma-Aldrich) and 2.5 μg/ml AmphotericinB (Sigma-Aldrich). Primary melanocytes were isolated from unaffected choroid, obtained at the time of enucleation. Normal uveal melanocytes were handled in the same manner as primary uveal melanoma cells except they were maintained in OPTI-MEM medium supplemented with 10 ng/ml bFGF (PeproTech Inc, Rocky Hill NJ, USA), 10 ng/ml PMA (Sigma-Aldrich), 0.1 mM IBMX (Sigma-Aldrich), 1 ng/ml Heparin (Sigma-Aldrich), 50 μg/ml Gentamicin (Sigma-Aldrich) and 2.5 μg/ml AmphotericinB (Sigma-Aldrich).

### BAP1 depletion

Transient knockdown was carried out using BAP1 or control siRNA (Ambion, Austin TX, USA) in 92.1 and Mel290 uveal melanoma cell lines as previously described [3]. Lentiviral-based short hairpin RNA (shRNA) was used to deplete BAP1 or control gene, GFP from cultured cells for long-term experiments. Lentiviral pLKO.1 shRNA vectors for GFP (clonetechGfp-438s1c1) and BAP1 (NM_004656.2-2658s1c1 and NM_004656.2-321s1c1) developed by the RNAi Consortium (TRC)] were purchased from the Children's Discovery Institute/Genome Sequencing Center at Washington University in St. Louis. Viral production and infections were carried out according to The RNAi Consortium recommendations (Broad Institute). Lentiviruses were packaged in 293FT cells (Invitrogen, Carlsbad CA, USA) after cotransfection of the shRNA plasmids with pCMV-dR8.2 dvpr and pCMV-VSV-G lentiviral plasmids (Addgene plasmids #8454 and #8455, deposited by Bob Weinberg, [21]) using TransIT-LT1 (Mirus, Madison WI, USA). Cells were infected for 24 hours with lentiviral supernatants in the presence of 5 μg/ml protamine sulfate. Puromycin (3 μg/mL) was added to the cells at 24 hours postinfection for selection as previously described [16]. With the exception of primary class 1 tumor cells, which were under selection for one week, all infected cells were selected for at least two weeks before use in experiments and were maintained under selection for up to four weeks (referred to as BAP1-deficient or control stable cells).

### Growth assays

MTS assays were performed using CellTiter 96 AQueous Assay reagent (Promega, Madison WI, USA) according to manufacturer's instructions. Bromodeoxyuridine (BrdU) incorporation assays were performed in 96-well plates and colorimetric changes were measured at 370 nm using a Microplate spectrophotometer (SpectraMAX 190; Molecular Devices) as previously described [16]. Flow cytometry was performed using a standard propidium iodide staining protocol as previously described [22] using a FACScan analyzer (BD Biosciences, San Diego CA, USA). The percentage of cells in each phase was determined using FlowJo software. Assays assessing the growth of cells in stem cell conditions were performed by plating 1000 or 2000 cells/well (OCM1A or 92.1, respectively) in 24-well ultra-low attachment plates containing stem cell medium, MDMF. After 5 or 7 days (OCM1A or 92.1, respectively) images were taken at 40X magnification and colony size was measured using ImageJ software. For clonogenic assays using OCM1A and 92.1 cells, flow cytometry (MoFlo; Cytomation) was used to seed one viable cell per well in ultra-low attachment 96-well plates containing MDMF medium as previously described [23]. Cell morphology of primary uveal melanocytes was assessed by phase contrast microscopy as previously described [16].

For HDAC inhibitor studies 0.5 mM, 1.0 mM or 2.0 mM valproic acid (Sigma-Aldrich) dissolved in water was added to BAP1-deficient or control cells for 72 hrs before RNA was isolated.

### Tumorigenicity assays

Soft agar assays were performed as previously described [23]. Plates were stained with MTT after 2 weeks and images were taken 6.7X using a dissecting scope and colonies were counted using ImageJ software. Scratch assays were carried out by plating 2x10^5^ cells/well in 12 well plates. Before scratching with a P200 tip, cells were treated with 5 μg/ml mitomycin C for 2 hrs at 37°C and washed with PBS. Two 100X images were taken per well and a total of three wells were imaged per condition for each experiment. Images were taken at Day 0, 1 and 2 and closure of the scratch was measured using ImageJ. Time-lapse microscopy was performed by plating cells on collagen coated 8-well chamber slides (Thermo Fisher Scientific, Rochester NY, USA) at a concentration of 1000 cells/well. The cells were allowed to attach overnight at 37°C and then imaged using an inverted Nikon Eclipse Ti at 200X every 15 minutes for 16 hrs. Cells were manually tracked using NIS Elements software (Nikon, Melville NY, USA).

### Immunoprecipitations and western blots

Cell lysates for both westerns and immunoprecipitations (IPs) were prepared by resuspending cell pellets in lysis buffer, which consists of 50 mM Hepes pH7.2, 400 mM NaCl, 0.1% NP-40, 0.5 mM EDTA pH8, 2.5 mM DTT, plus protease and phosphatase inhibitors. Samples were then incubated on ice for 10mins before a 10 sec, low-power sonication. After which, samples were spun down to remove cellular debris and supernatants were then used for either westerns or IPs. For westerns 20 μg of protein was loaded for each sample. IPs were performed using combined lysates from OCM1A, 92.1, and Mel290 uveal melanoma cell lines. After sonication, lysates were pre-cleared with ProteinG Sepharose beads (Sigma-Aldrich) for 1 hr and incubated overnight at 4°C with 5 μg of the indicated antibodies. After incubation for 1 hr with fresh sepharose beads, samples were spun down and beads were washed twice with lysis buffer. Proteins were eluted by boiling the samples with 6X SDS loading buffer for 5 mins. IP supernatants (the supernatant removed from the tube after the initial IP pulldown) were kept for western blot analysis and are referred to as cleared lysates. IP samples and cleared lysates were subjected to SDS-PAGE followed by western blotting for the indicated antibodies. Densitometry was performed on western blots using ImageJ software. Antibodies used for IP and western blot were BAP1 (Santa Cruz, Santa Cruz CA, USA), HCF-1 (Bethyl Laboratories, Montgomery TX, USA), α-tubulin (Sigma-Aldrich), and control antibodies rabbit IgG and mouse IgG (Santa Cruz).

### RNA analysis

For primary melanocytes and tumor samples total RNA was extracted with TRIzol (Invitrogen) according to the manufacturer’s protocol and purified by ammonium acetate precipitation. RNA was extracted from cell lines using an RNeasy Kit (Qiagen) according to the manufacturer’s protocol. The RNA was DNase treated and reverse transcribed using iScript cDNA Synthesis Kit (Bio Rad, Hercules CA, USA). Primary melanocyte and tumor sample RNA was preamplified for 14 cycles with pooled primers according to the manufacturer's protocol using TaqMan PreAmp Master Mix (Applied Biosystems, Foster City CA, USA). mRNA levels were measured by qPCR using iTaq SYBR Green Supermix (Bio Rad) as previously described [3]. UBC was used as an endogenous control. Primer sequences are listed in Additional file 1.

### Gene expression profiling

Gene expression profiling (GEP) was performed on two independent sets of uveal melanoma cell lines (OCM1A, 92.1 and Mel290), each expressing either GFP or BAP1 shRNA for four weeks (12 samples total). Total RNA was isolated using the RNeasy kit (Qiagen). RNA quality was assessed on the Bioanalyzer 2100 (Agilent Technologies, Santa Clara, CA, USA). Samples were subjected to gene expression profiling using the HumanHT-12 v4 Expression BeadChip (Illumina, San Diego, CA, USA). Raw expression data were subjected to cubic spline normalization in GenomeStudio (version 2011.1). ANOVA and hierarchical clustering were performed with Partek Genomics Suite (version 6.6) using a significance of *P* < 0.01 as a threshold for gene inclusion. Significance Analysis of Microarrays (SAM), Version 4.0 [24] was used to generate a ranked gene list, and a threshold of q < 10% was then used to select the most highly significant genes that were up or down regulated after BAP1 loss. This list was used to determine the most highly represented gene ontology categories and genes from this list were selected for validation by qPCR. A pre-ranked file was generated from the SAM output data and run through Gene Set Enrichment Analysis (GSEA), version 2.0.4 [25,26] to identify significantly enriched gene sets. Gene expression data have been deposited in the NCBI Gene Expression Omnibus and are accessible through GEO Series accession number GSE48863.

### SNP arrays

Three uveal melanoma cell lines (OCM1A, 92.1, and Mel290) expressing either GFP or BAP1 shRNA for four weeks were subjected to single nucleotide polymorphism (SNP) arrays using Affymetrix Human Genome-Wide SNP 6.0 array (Affymetrix, Santa Clara, CA, USA). DNA was isolated using a DNeasy kit (Qiagen). Copy number and allele ratios were calculated using Partek Genomics Suite (version 6.6). For paired analyses, cell lines expressing GFP shRNA were used as baselines; for unpaired analyses, the Partek distributed baseline was used as a reference. Hidden Markov Model genomic smoothing was used to identify significant regions of amplification and deletion in samples expressing BAP1 shRNA compared to control samples.

### Animal studies

Animal experiments were approved by the Washington University in St. Louis Animal Studies Committee. 5–8 week old NOD.Cg-Prkdc^scid^ Il2rg^tm1Wjl^/SzJ JAX [non-obese diabetic severe-combined immunodeficient gamma (NSG)] males (Jackson Laboratory) were injected subcutaneously into the flank with 500 OCM1A or 1000 92.1 cells in 50 μl Cultrex (Trevigen, Gaithersburg MD, USA). Tumor size was monitored once a week and the mice were euthanized after 34 (OCM1A) or 64 (92.1) days at which time tumors were collected and measured. Volume of each tumor was calculated using the ellipsoid volume formula (π xyz/6 mm^3^). Tumors were collected in TRIzol at time of necropsy for RNA isolation. For different experiments 10,000 92.1 cells or 500,000 OCM1A were injected into the tail vein of 5–8 weeks old NSG males or females. Mice were monitored and euthanized after 29 or 44 days respectively. Organs were collected and fixed in 10% formalin. Fixed liver and lungs were cut into 5 mm thick pieces, dehydrated and embedded in paraffin as a single block. Four micron sections were cut and stained with H&E. Overlapping images of the sections were taken at 20X and merged to one image using AdobePhotoshop CS4. Total liver or lung area, and metastasis area were measured using ImageJ 1.45 s for calculation of % of metastasis.

## Results

### BAP1 loss causes transient cell cycle inhibition

**Figure 1:**
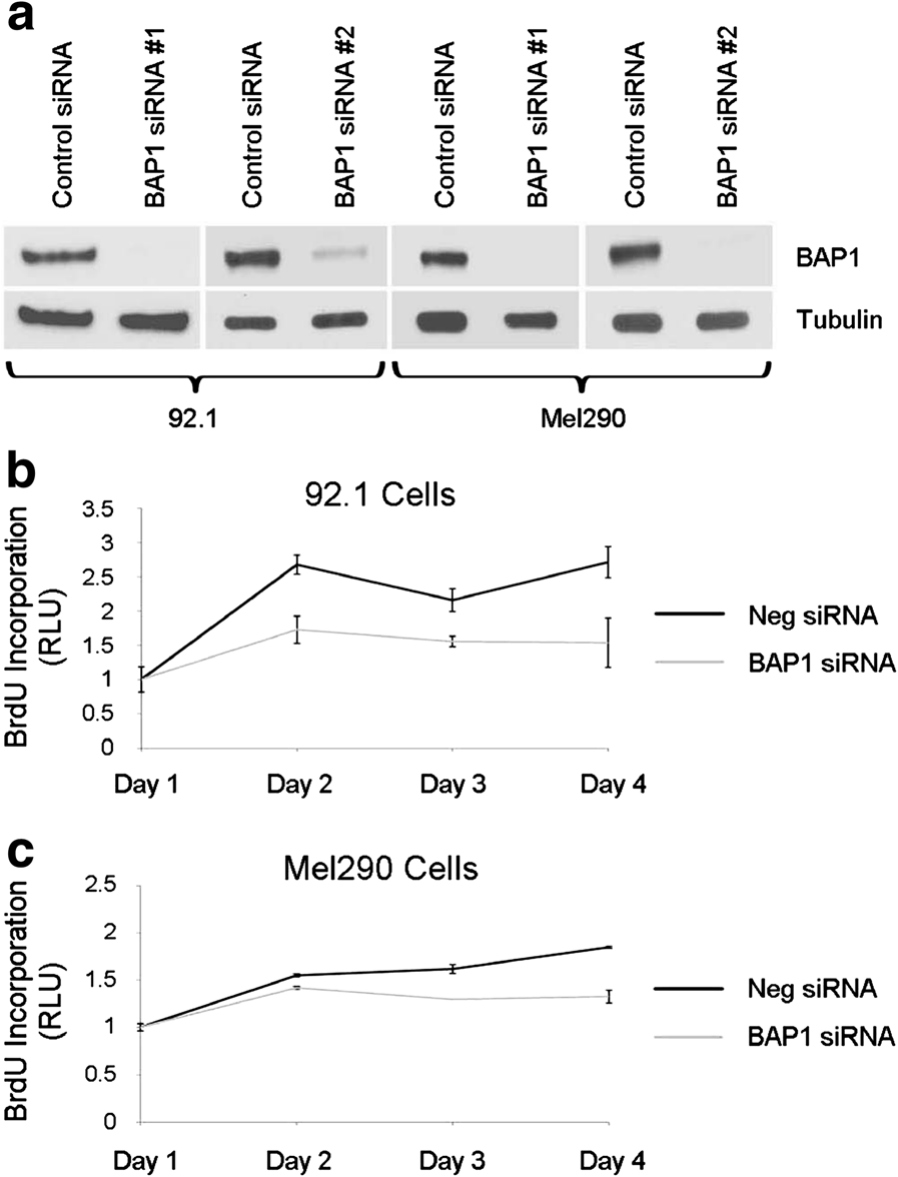
**Transient loss of BAP1 causes a decrease in proliferation in uveal melanoma cells. (a)** Western blot of two uveal melanoma cell lines, 92.1 and Mel290, showing the levels of BAP1 after transient knockdown of BAP1 with two independent siRNAs compared to control siRNA. α-tubulin was used as a loading control **(b-c)** Cell proliferation of the indicated melanoma cell lines transfected with BAP1 (grey line) or control (black line) siRNA. Proliferation was measured by BrdU incorporation and shown as fold change compared to Day 1 **(b)** 92.1 cells **(c)** Mel290 cells.

**Figure 2:**
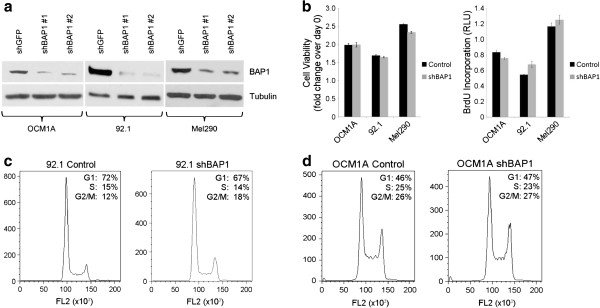
**Stable loss of BAP1 does not promote cell proliferation in uveal melanoma cells. (a)** Western blot showing the levels of BAP1 after stable knockdown of BAP1 with two independent lentiviral shRNA constructs in three uveal melanoma cell lines, OCM1A, 92.1, and Mel290. α-tubulin was used as a loading control **(b)** Cell viability and proliferation of the indicated BAP1-deficient or control stable cells (Left panel) cell viability measured by MTS assay and shown as fold change over day 0 for each stable cell line (Right panel) cell proliferation measured by BrdU incorporation after 24 hrs **(c-d)** Cell cycle analysis of the indicated cell lines using flow cytometry on propidium iodide stained cells; x-axis represents DNA content and y-axis represents cell number **(c)** 92.1 stable cells **(d)** OCM1A stable cells.

To study the effects of BAP1 loss in uveal melanoma cells, we initially used siRNA to achieve at least an ~ 80% depletion of BAP1 protein levels (Figure 1a). This resulted in a 20-40% reduction in cell cycle progression, measured by BrdU incorporation in two different uveal melanoma cell lines (92.1 and Mel290), which persisted through the four day experiment (Figure 1b-c). This observation is consistent with previous findings in MCF10A breast epithelial cells and HeLa cervical carcinoma cells [11,13,27]. To study longer-term effects of BAP1 loss, we used short-hairpin RNA (shRNA) expressed from lentiviral vectors, which consistently achieved 70-90% depletion of BAP1 protein levels in three different uveal melanoma cell lines (OCM1A and 92.1, and Mel290) (Figure 2a). BAP1-depleted cells were then compared to those infected with control lentivirus expressing shRNA directed against GFP. Interestingly, there was no substantial difference in cell viability, BrdU incorporation or cell cycle profile between BAP1-deficient and control cells after stable expression of the shRNA constructs for at least 14 days (Figure 2b-d), indicating that the initial cell cycle inhibition caused by BAP1 depletion was transient.

### Effects of BAP1 loss on tumorigenicity

**Figure 3:**
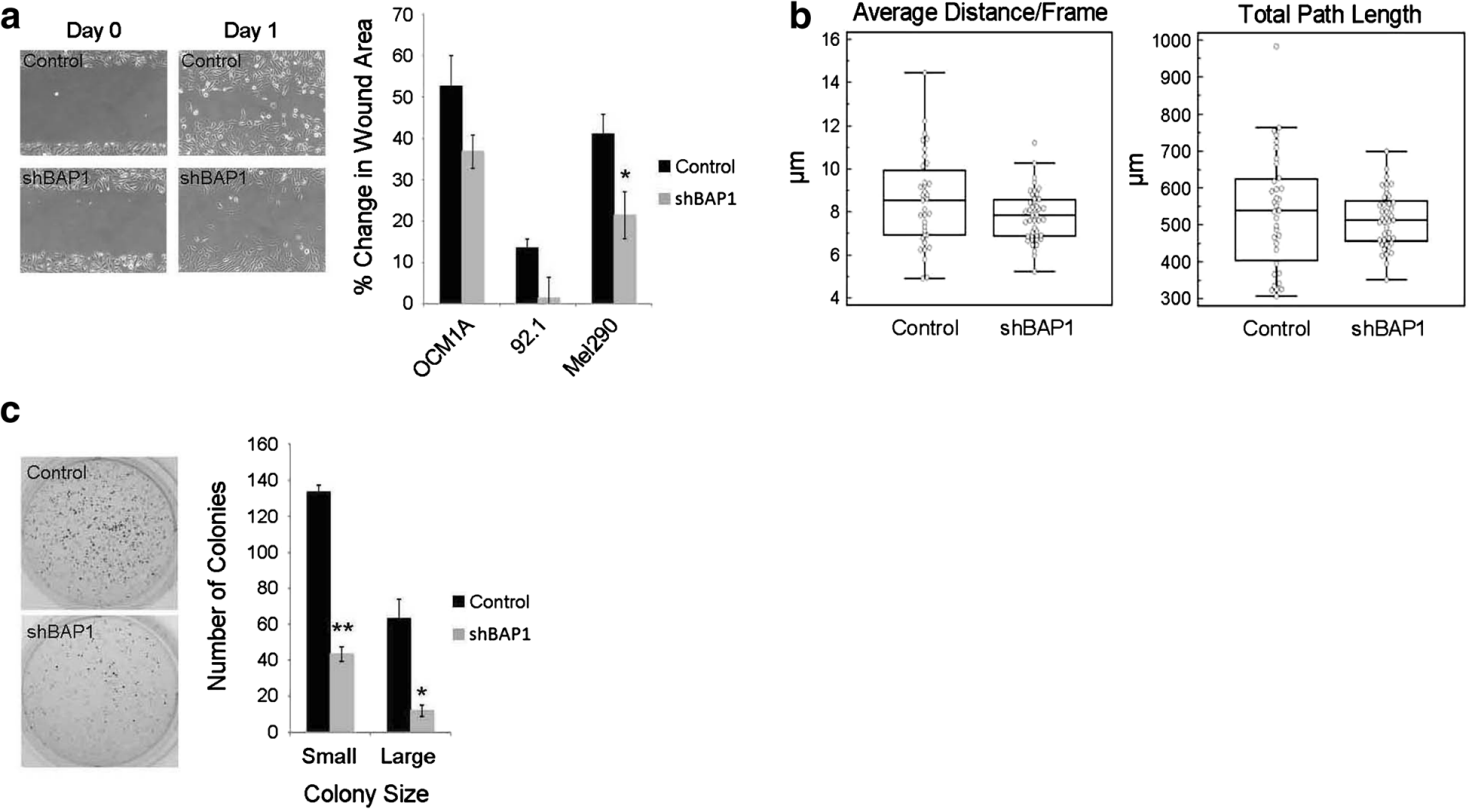
**Loss of BAP1 does not promote *in vitro *tumorigenicity in uveal melanoma cells. (a)** Wound healing assays were performed on the indicated BAP1-deficient or control stable cells. (Left panel) representative pictures of Mel290 cells after stable knockdown at Day 0 (time of scratch) and Day 1. (Right panel) quantification of wound healing assays shown as percent of total area of the initial wound **(b)** BAP1-deficient or control Mel290 stable cells were monitored every 15mins for 16 hrs using live cell imaging. Individual cells were manually tracked and the average distance per frame (left panel) and the total distance traveled (right panel) were calculated **(c)** Soft agar assays performed on BAP1-deficient or control OCM1A stable cells after one week of growth. (Left panel) representative pictures of soft agar plates stained with crystal violet. (Right panel) quantification of the number of small and large colonies per plate determined using ImageJ software. * denotes *P* < 0.05 and ** denotes *P* < 0.01 based on Student’s t-test.

The uveal melanoma cells stably expressing shRNA against BAP1 and control shRNA against GFP were compared using *in vitro* and *in vivo* assays of tumorigenicity. Using scratch assays as a measure of cell motility, BAP1-deficient uveal melanoma cells were less motile than control cells (Figure 3a). Prompted by this unexpected finding, we performed time-lapse microphotography and confirmed that BAP1-deficient cells showed less overall movement than control cells (Figure 3b). Similarly, BAP1-deficient uveal melanoma cells were less capable than control cells of anchorage independent growth in soft agar assays (Figure 3c).

**Figure 4:**
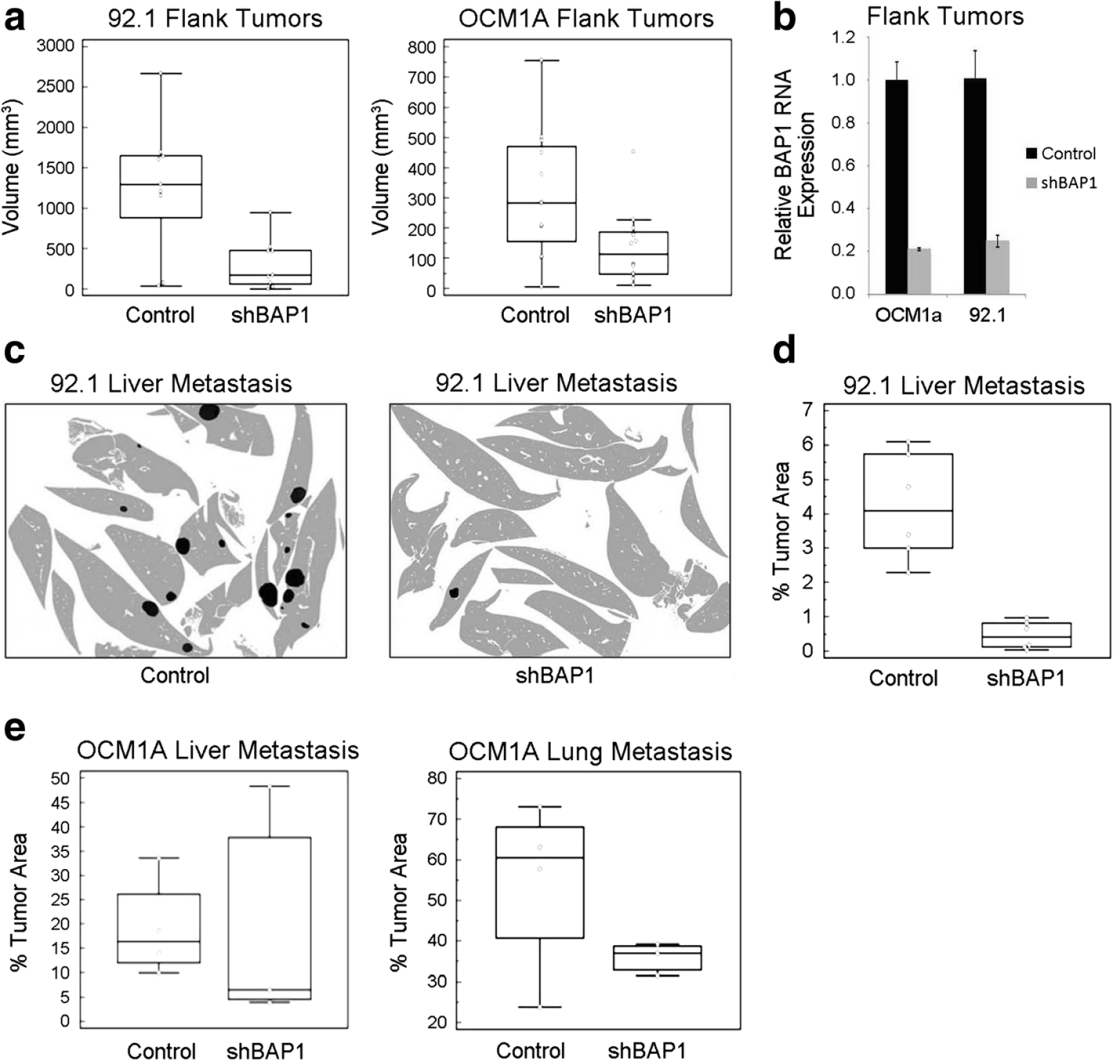
**Loss of BAP1 does not promote growth of uveal melanoma in mouse flank and tail vein xenograft models. (a)** Mouse xenograft flank injections in which 1000 or 500 BAP1-deficient or control 92.1 or OCM1A stable cells, respectively were injected into the flanks of NSG mice. (Left panel) 92.1 stable cells (Right panel) OCM1A stable cells. Tumor volume was measured at time of necropsy 64 days or 35 days after injection for 92.1 and OCM1A cells, respectively. **(b)** RNA expression levels of BAP1 in flank tumors formed from BAP1-deficient of control stable cell lines. RNA was isolated at time of necropsy. Expression is shown as fold change compared to control shRNA cells **(c-d)** Mouse xenograft tail vein injections in which 10,000 BAP1-deficient or control 92.1 stable cells were injected into the tail veins of NSG mice. Necropsy was performed 29 days after injection and liver metastasis was assessed **(c)** Representative cartoons of liver metastasis made from merged images of liver sections stained with H&E. Images were merged using Adobe Photoshop. Normal liver tissue is shown in grey and metastases are in black. Original H&E merged images are shown in Additional file 2. **(d)** Quantification of liver metastasis. Shown as the percent of tumor area compared to the total liver area. ImageJ software was used to calculate area **(e)** Mouse xenograft tail vein injections in which 500,000 BAP1-deficient or control OCM1A stable cells were injected into the tail veins of NSG mice. Liver and lung metastases were measured 30 days after injection. Metastases are shown as a percent of the total liver or lung area.

To assess the ability to form tumors *in vivo*, we created flank tumors in NOD-SCID gamma mice using BAP1-deficient versus control uveal melanoma cells. Surprisingly, the BAP1-deficient tumors were smaller than control tumors (Figure 4a). We confirmed that BAP1 was still depleted in these tumors by isolating RNA at the time of necropsy and performing qPCR (Figure 4b). To assess metastatic capacity, we then performed tail vein injections of BAP1-deficient and control uveal melanoma cells in the same mouse strain, and the BAP1-deficient cells formed fewer metastases in the liver and lungs compared to control cells (Figure 4c-e and Additional file 2).

### Global genomic effects of BAP1 loss

**Figure 5:**
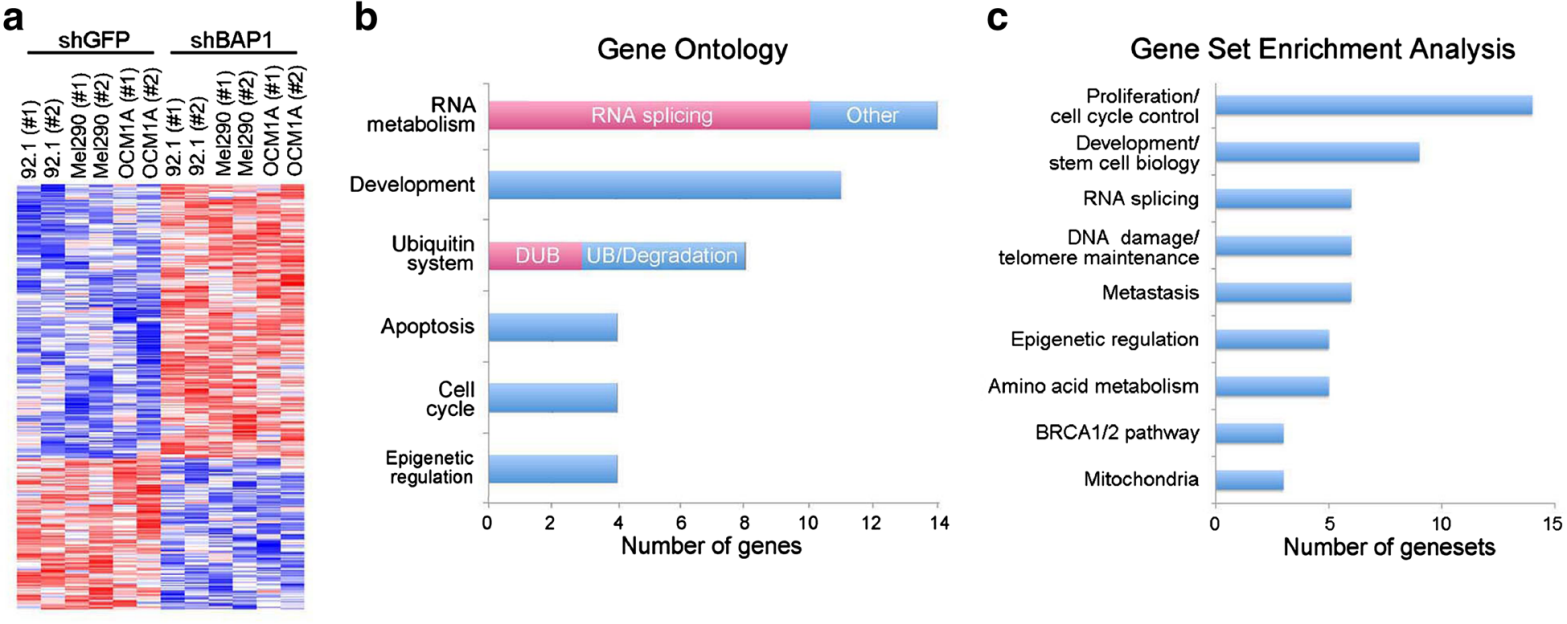
**Gene expression profiling reveals altered RNA metabolism and developmental transcriptomic signatures in BAP1-deficient uveal melanoma cells. (a)** Gene expression heatmap generated in Partek of significantly altered genes (*P* < 0.01) in BAP1-deficient stable cells compared to control stable cells after 4 weeks of shRNA expression. **(b)** The top six gene ontology categories represented by the significantly changing genes in BAP1-deficient cells. Significant Analysis of Microarrays (SAM) was performed and a cutoff of q < 10% was used to determine the most highly significant genes. Three of the genes in the ubiquitin system category are deubiquitinases (DUBs). **(c)** The top nine categories represented after gene set enrichment analysis (GSEA) of BAP1-deficient cells using a threshold of *P* < 0.005. GSEA was performed using a pre-ranked file generated after SAM analysis.

Given these unexpected findings, we wished to gain insights into the role of BAP1 loss in uveal melanoma progression by analyzing the changes in global gene expression associated with BAP1 depletion. We analyzed the transcriptome of all three uveal melanoma cell lines using Illumina BeadArrays at four weeks after stable shRNA expression (Figure 5a). In order to identify the most significantly altered genes, we used Significance Analysis of Microarrays (SAM) with a false discovery rate cut-off of 10% and found 77 genes that were up-regulated (16 of these were pseudogenes or uncharacterized loci), and 6 genes that were down-regulated by BAP1 depletion (Additional file 3 and Additional file 4). The finding that more genes were up-regulated than down-regulated by depletion of BAP1 is consistent with its known role in transcriptional repression as part of the Polycomb PR-DUB complex [14]. The most common Gene Ontology categories included RNA metabolism (14 genes, including 10 specifically involved in RNA splicing), developmental processes (11 genes), ubiquitin system (8 genes), apoptosis (4 genes), cell cycle (4 genes), and epigenetic regulation (4 genes) (Figure 5b). Among the genes involved in the ubiquitin system, three were involved not with ubiquitin-associated protein degradation, but with substrate deubiquitination.

The set of differentially expressed genes was further analyzed for functional significance using Gene Set Enrichment Analysis. Genes with altered expression upon BAP1 depletion exhibited significant (p < 0.005) enrichment in gene sets involved in proliferation/cell cycle control (14 gene sets), development and stem cell bio-logy (9 gene sets), RNA splicing (6 gene sets), DNA damage repair (6 gene sets), metastasis (6 gene sets), epigenetic regulation (5 gene sets), amino acid metabolism (5 gene sets), the BRCA1/2 pathway (3 gene sets) and mitochondrial activity (3 gene sets) (Figure 5c and Additional file 5, Additional file 6 and Additional file 7). The metastasis gene sets included genes that were up-regulated in metastasizing melanoma, as well as prostate, lung, and pancreatic cancer.

There were 3 BRCA1/2 pathway gene sets indentified, and among the 6 DNA damage repair gene sets, 3 were related to telomere maintenance. Since BRCA pathway deregulation and telomere dysfunction are both associated with amplifications and deletions in cancer cells [28,29], we wished to determine whether BAP1 depletion might result in such large-scale chromosomal gains and losses in uveal melanoma cells. However, Affymetrix 6.0 SNP arrays showed no differences in chromosome number between BAP1-deficient versus control cells for any of the three uveal melanoma cell lines after 4 weeks of BAP1 depletion. (Additional file 8).

### BAP1 loss induces a stem-like cellular phenotype in melanoma cells

**Figure 6:**
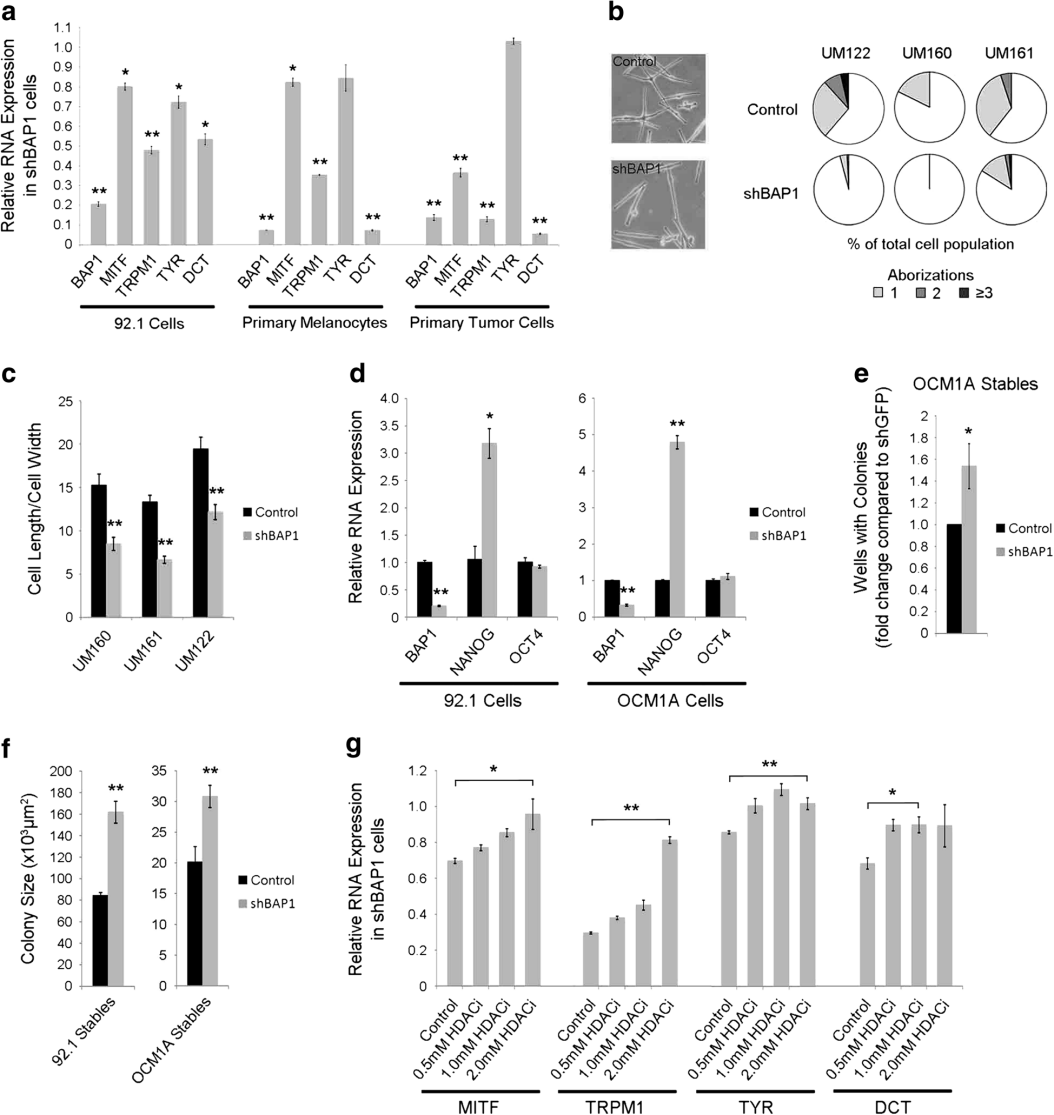
**Loss of BAP1 induces a dedifferentiated, stem-like phenotype in uveal melanoma cells. (a)** RNA expression of melanocyte differentiation markers in BAP1-deficient or control 92.1 stable cells, primary melanocytes, and primary class 1 tumor cells. Fold change is compared to control knockdown cells. **(b)** Cell morphology of BAP1-deficient or control primary uveal melanocytes. (Left panel) representative pictures. (Right panel) dendritic arborizations measured as the number of branch points per cell, with a bipolar cell having zero aborizations; shown as percent of total cells. **(c)** Cell spindle morphology of BAP1-deficient or control primary uveal melanocytes after four weeks of shRNA expression, measured as the ratio of cell length to cell width. **(d)** RNA expression levels of stem cell genes NANOG and OCT4 in BAP1-deficient or control 92.1 and OCM1A stable cells. Fold change is compared to control knockdown cells. **(e)** Clonogenic assay using BAP1-deficient or control OCM1A stable cells. One cell/well was grown in serum-free stem cell media in non-adherent 96-well plates. The number of wells producing colonies was measured after 5 days. Data is shown as fold change over control. **(f)** Assay measuring the ability of BAP1-deficient or control stable cells to grow in stem cell culture conditions on non-adherent plates in serum-free media. Colony size was measured after 5 (OCM1A) or 7 (92.1) days using ImageJ software. **(g)** RNA expression of melanocyte differentiation markers in BAP1-deficient 92.1 stable cells after 72 hr treatment with 0.5 mM, 1.0 mM, or 2.0 mM HDAC inhibitor (HDACi) as indicated. Fold change is compared to control knockdown cells.

Prompted by these transcriptomic findings, we wished to explore further the possibility that BAP1 inhibits metastasis of uveal melanoma cells by maintaining their differentiated state and impeding their reversion to a stem-like state. Consistent with this hypothesis, depletion of BAP1 caused a down-regulation of canonical genes of the melanocyte differentiation program (*MITF, TRPM1, TYR and DCT*) (Figure 6a). Similar changes were seen in cultures of primary uveal melanocyte samples from three independent patients stably expressing shRNA against BAP1 or control shRNA against GFP and also in two short-term cultures from fresh primary class 1 tumors (Figure 6a and data not shown). Further, stable depletion of BAP1 in cultured primary uveal melanocytes resulted in cells with fewer dendritic aborizations and less differentiated spindle morphology, both of which suggest melanocyte dedifferentiation (Figure 6b-c). In addition, we saw consistent up-regulation of the stem cell factor NANOG in BAP1-depleted uveal melanoma cells (Figure 6d). OCT4 expression did not change with BAP1 depletion, but this stem cell factor is tightly maintained within a restricted range to avoid differentiation [30].

To assess the capacity for self-replication, which is a measure of “stemness”, BAP1-deficient and control cells were flow sorted, single cells were seeded into separate wells of low-attachment 96-well plates in serum-free stem cell media, and the presence or absence of colonies from each well was assessed at 5 days. The BAP1-deficient cells exhibited a 50% increased capacity for self-replication compared to control cells (Figure 6e). Further, whereas we showed earlier that BAP1-deficient cells produced colonies in soft agar less efficiently than control cells using our usual serum-containing culture media (Figure 3c), the BAP1-deficient cells grew more efficiently than control cells in the limiting stem cell conditions of serum-free media and low attachment plates (Figure 6f and Additional file 9).

As we showed previously, HDAC inhibition reverts primary class 2 uveal melanoma cells to a differentiated, less aggressive class 1 phenotype [16]. Consistent with those results, treatment of BAP1-deficient uveal melanoma cells with an HDAC inhibitor restored the expression of the melanocyte differentiation markers, which were down-regulated by BAP1 depletion, in a dose-dependent manner (Figure 6g).

**Figure 7:**
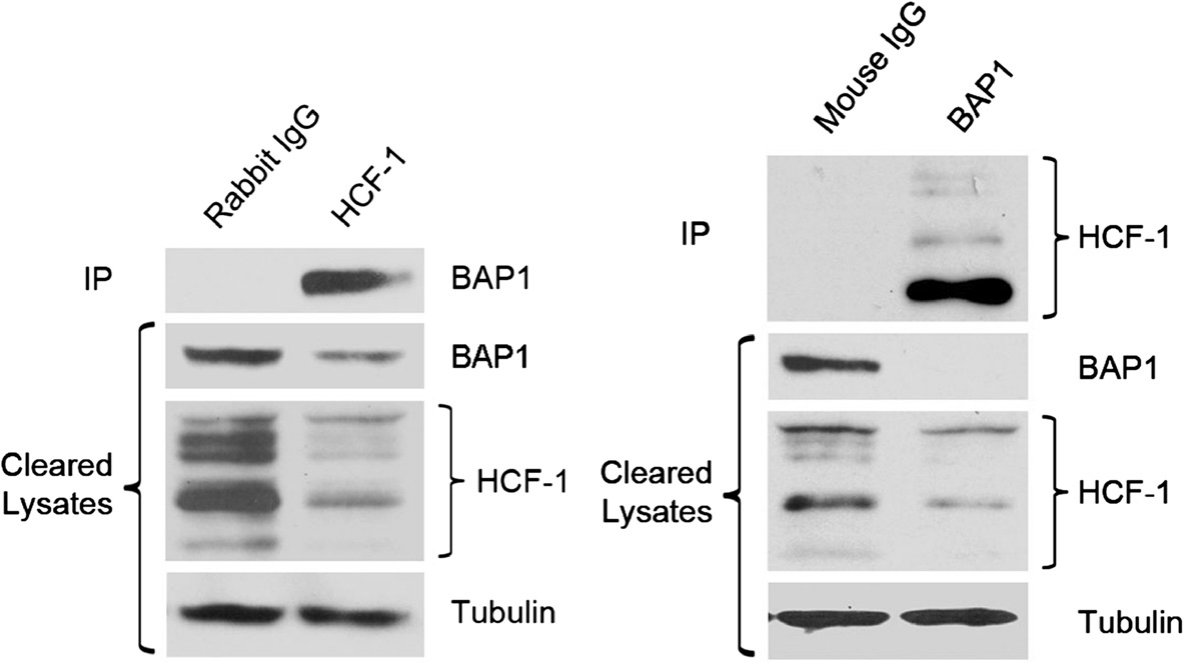
**Physical interaction between BAP1 and HCF-1 in uveal melanoma cells.** Immunoprecipitations (IP) for endogenous BAP1 and HCF-1 from the combined lysates of UM cell lines. Westerns were performed on IP material and lysate supernatant that was collected after the IP (cleared lysates). α-tubulin was used as a loading control for the cleared lysate samples only. Densitometry was performed using ImageJ and showed ~75% of the total BAP1 was complexed with HCF-1. * denotes *P* < 0.05 and ** denotes *P* < 0.01 based on Student’s t-test.

A major binding partner of BAP1 protein is the transcriptional co-regulator HCF-1, which was recently shown to play a key role in stem cell maintenance, in part through regulation of RNA splicing [31]. As this interaction has not been addressed within the melanocytic lineage, we examined the interaction between endogenous BAP1 and HCF-1 in BAP1-wildtype uveal melanoma cells. Indeed, HCF-1 and BAP1 were found to co-precipitate using antibodies against either protein for immunoprecitapation, and approximately 75% of total cellular BAP1 was in a complex with HCF-1 (Figure 7).

## Discussion

In this report, we studied the effects of both transient and stable RNAi-mediated depletion of BAP1 in uveal melanoma cells. In agreement with previous work in other cell types [11,13,27], transient BAP1 loss decreased cell proliferation. However, we now show that this effect is not sustained in cells that are stably depleted of BAP1, implying that this initial growth defect is not a critical factor in BAP1’s role as a tumor suppressor. Despite variable levels in knockdown with the different siRNAs and shRNAs used, we saw no correlation between the level of expression and the degree of proliferation defect. Surprisingly, stable loss of BAP1 had minimal or even paradoxical effects in most standard assays of tumorigenicity, including cell cycle control, motility and the ability to form colonies in soft agar, suggesting that BAP1 loss promotes tumor progression in a manner that is different from most characterized tumor suppressors.

The most striking effect of BAP1 loss was the induction of a primitive, stem-like phenotype characterized by a loss of morphologic differentiation, down-regulation of the melanocyte transcriptional program, up-regulation of genes enriched in stem cells and developmental processes, and enhanced growth capacity under stem cell conditions. These findings are consistent with our previous findings in class 2 primary uveal melanomas *in vivo*[32], and they implicate BAP1 in the maintenance of cell identity in uveal melanoma.

Our findings are also in agreement with other recent work on BAP1 function. BAP1 is a component of the PR-DUB Polycomb repressive complex, which catalyzes the removal of monoubiquitin moieties from H2A in opposition to the ubiquitinating activity of the PRC1 complex that contains BMI1 [14]. We recently showed that HDAC inhibitors, which block BMI1, revert primary class 2 uveal melanoma cells to a differentiated class 1 phenotype. We now go on to show that HDAC inhibitors restore to normal levels the expression of melanocyte differentiation genes that are down-regulated by BAP1 depletion. Our work suggests that BAP1 activity is important for maintaining melanocytic cell identity.

The transcriptional co-regulator HCF-1 is a major binding partner of BAP1 and may regulate the genomic localization of BAP1 through a multi-protein interaction with the transcription factor YY1 [12,13] or, as shown more recently, through interactions with OGT and FOXK1/2 [17]. HCF-1 has traditionally been thought of as a cell cycle regulator, but it now appears that the complexes in which HCF-1 is found while regulating the cell cycle may be distinct from those in which BAP1 is found [17]. HCF-1 plays a key role in stem cell maintenance, at least in part by regulating genes involved in RNA splicing [31], and we showed here that HCF-1 is the predominant BAP1 binding partner in uveal melanoma cells, and that genes regulated by BAP1 are enriched for those involved in cell cycle control and RNA splicing and processing. Further work is needed to clarify the precise mechanism of action of BAP1 and HCF-1 in tumor suppression, which may vary depending on context and cell type.

Gene Set Enrichment Analysis of transcripts that were deregulated in cells depleted of BAP1 revealed enrichment of gene sets associated with metastasis in melanoma, prostate, lung, and pancreatic cancer, suggesting a more general role for BAP1 loss in cancer progression. These transcripts were also enriched in gene sets related to the ubiquitin system, including both proteasomal and chromatin remodeling components. This is consistent with a growing body of work showing that these two components of the ubiquitin system are in a dynamic equilibrium that balances a rate-limiting pool of free ubiquitin [33]. These genes were also enriched in 6 DNA damage/telomere maintenance gene sets and in 3 gene sets that were specifically related to the BRCA1/2 pathway. This is consistent with previous work linking BAP1 to BRCA1 [10]. However, the fact that BRCA1 was not identified as a BAP1-interacting protein in several unbiased screens in different cell types [12,18] indicates that the cellular context in which this interaction may be relevant remains unclear. One possibility is that BRCA1 and BAP1 interact specifically in the setting of DNA damage repair, where H2A is monoubiquitinated by BMI1, and BRCA1 is recruited to DNA lesions [34,35]. In any event, it is likely that the effects of BAP1 loss are likely to be cell type-specific and context-dependent.

The precise mechanism by which the loss of cell identity induced by BAP1 loss leads to metastasis remains unclear. The fact that BAP1-depleted uveal melanoma cells did not exhibit a growth advantage or increased metastatic capacity in xenograft mouse models was surprising but indicates that these models are not adequate for elucidating the role of BAP1 *in vivo*. One possibility is that the genetic and/or epigenetic mechanisms that prevent uveal melanocytes, which are derived from the migratory cranial neural crest, from migrating away from the eye may be disrupted by the loss of cell identity. If this were the case and the critical event triggered by BAP1 loss was the escape of tumor cells from the eye, then our available xenograft models may be insufficient to model this. Further investigation of this issue will await the availability of genetically engineered animals models.

## Conclusions

In summary, we demonstrate that BAP1 is necessary for maintenance of melanocyte identity in uveal melanoma cells, and that loss of BAP1 leads to a loss of cell identity and acquisition of a primitive, stem-like phenotype. This effect is very similar to overexpression of the BAP1 antagonist, BMI1 in many forms of cancer [15] and points out the vital role of histone ubiquitination and Polycomb-mediated chromatin remodeling in cancer progression. Therapeutic strategies that target these pathways are urgently needed.

## Competing interests

JWH, AMB and Washington University may receive income based on a license of related technology by the University to Castle Biosciences, Inc. This work was not supported by Castle Biosciences, Inc.

## Authors’ contributions

KAM carried out all *in vitro* experiments and participated in *in vivo* experiments, data analysis and writing the manuscript. OAA performed *in vivo* experiments. MDO performed microarray data analysis. LAW collected and maintained human samples. AMB participated in conceiving the project and with manuscript preparation. JWH led the conception of the project and participated in writing the manuscript. All authors read and approved the final manuscript.

## Pre-publication history

The pre-publication history for this paper can be accessed here:

http://www.biomedcentral.com/1471-2407/13/371/prepub

## Supplementary Material

Additional file 1 **qPCR primer sequences.** A list of the forward and reverse primer sequences used in all qPCR reactions performed. Click here for file

Additional file 2 **Original H&E images of liver metastases.** The original merged H&E images of liver metastases after tail vein injection of 92.1 BAP1-deficient and control cells. Click here for file

Additional file 3 **SAM analysis of gene expression profile results.** Significant Analysis of Microarrays results showing genes that were up or down regulated in BAP1-deficient stable cells when compared to control cells. Only genes with a false discovery rate of less than 10% are shown. Click here for file

Additional file 4 Validation of select genes. qPCR validation in three uveal melanoma cell lines of select genes that were significantly altered after SAM analysis of gene expression profile results. Click here for file

Additional file 5 Top gene sets after GSEA analysis. A list of gene sets with a p < 0.005 after gene set enrichment analysis of BAP1-deficient cells. Click here for file

Additional file 6 **Enriched genes associated with each gene set category.** A list of genes enriched in at least two gene sets within a given category after GSEA analysis of BAP1-deficient stable cells when compared to control cells. The categories listed are those referred to in Figure 5c. Click here for file

Additional file 7 **Six of the top gene sets after GSEA analysis.** Six of the top gene sets significantly enriched in BAP1-deficient cells based on GSEA analysis. Click here for file

Additional file 8 **Single nucleotide polymorphism arrays.** Copy number analysis of single nucleotide polymorphism (SNP) arrays that were performed on three uveal melanoma cell lines (OCM1A, 92.1 and Mel290) expressing either BAP1 or control shRNA for four weeks. Click here for file

Additional file 9 **Representative images and MTS of stable cells in stem cell conditions.** (Left panels) Representative images of control and BAP1-deficient 92.1 stable cells after culture for 7 days in stem cell media in low attachment plates. (Right panel) MTS assay of control and BAP1-deficient 92.1 stable cells after culture for 7 days in stem cell media in low attachment plates. Click here for file
