# Supplementary material for: BAP1 deficiency causes loss of melanocytic cell identity in uveal melanoma
Source: BMC Cancer. 2013 Aug 5;13:371. doi: 10.1186/1471-2407-13-371 (PMC3846494; doi:10.1186/1471-2407-13-371)
Supplement: Additional file 1 — qPCR primer sequences. A list of the forward and reverse primer sequences used in all qPCR reactions performed. [file 1471-2407-13-371-S1.pdf]

Additional File 1. Primer sequences used in qPCR

Gene Name	Forward Primer Sequence	Reverse Primer Sequence
BAP1	GGTGGATGATACGTCCGTGAT	GCTCAGCAAGGCATGAGTTG
CKMT1A	CTCGAGACTGGCCAGATGCT	TCCATGGAGATCACCCGTGTA
DCT	TCAGTTTCAGGAATGCTTTGGAA	TCCCGTTCAGGAAGGAATGA
EXTL2	TCATCAGCACCCCAGACCTT	GATGAAGTAGAGACGTGCTTTCTAGGA
FAM175B	TGACTCACAAATCAGCAACACAGA	CATTCACTTTGCTTGCGTAGTCAT
HECTD2	TTCACCTGCACATCTTGTTTTCC	TGCATCCATAGATGTACGCTGTTT
MBNL1	ATCCGGGTGTCCCTGTACCT	GCAATTGCCACGTTGGTACTC
MITF	GAACTCAAAAGTCAACCGCTGAA	GCGTGATGTCATACTGGAGGAG
NANOG	TGCTTATTCAGGACAGCCCT	TCTGGTCTTCTGTTTCTTGACT
OCT4	GATAACTGGTGTGTTTATGTTCTTACAAGTCT	GCTTCAGGAGCTTGGCAAATT
PRPF4	GCAGGGATCGAAGCTGGAA	TCTCTCAAACTCAGCCAATACTTCTG
TNPO1	TATTGGTGACTCCTCTCCTCTGATTAG	TGGTAAGAGGTCAGGCCAATTC
TRPM1	ATGAGTTTCGGAGTAGCCCGTCAA	ATCTGGTCTGCAAACACCTCTCCA
TYR	ATTTTGCCTGAGTTTGACCCAA	CAGTAAGTGGACTAGCAAATCCTTCC
UBC	ATTTGGGTCGCGGTTCTTG	TGCCTTGACATTCTCGATGGT
UBE2K	GCTCGACTTTGGGCACATGT	TGCATTCCTATCAAAGCCCATAG
