# Supplementary figures and images for: BAP1 deficiency causes loss of melanocytic cell identity in uveal melanoma
Source: BMC Cancer. 2013 Aug 5;13:371. doi: 10.1186/1471-2407-13-371 (PMC3846494; doi:10.1186/1471-2407-13-371)

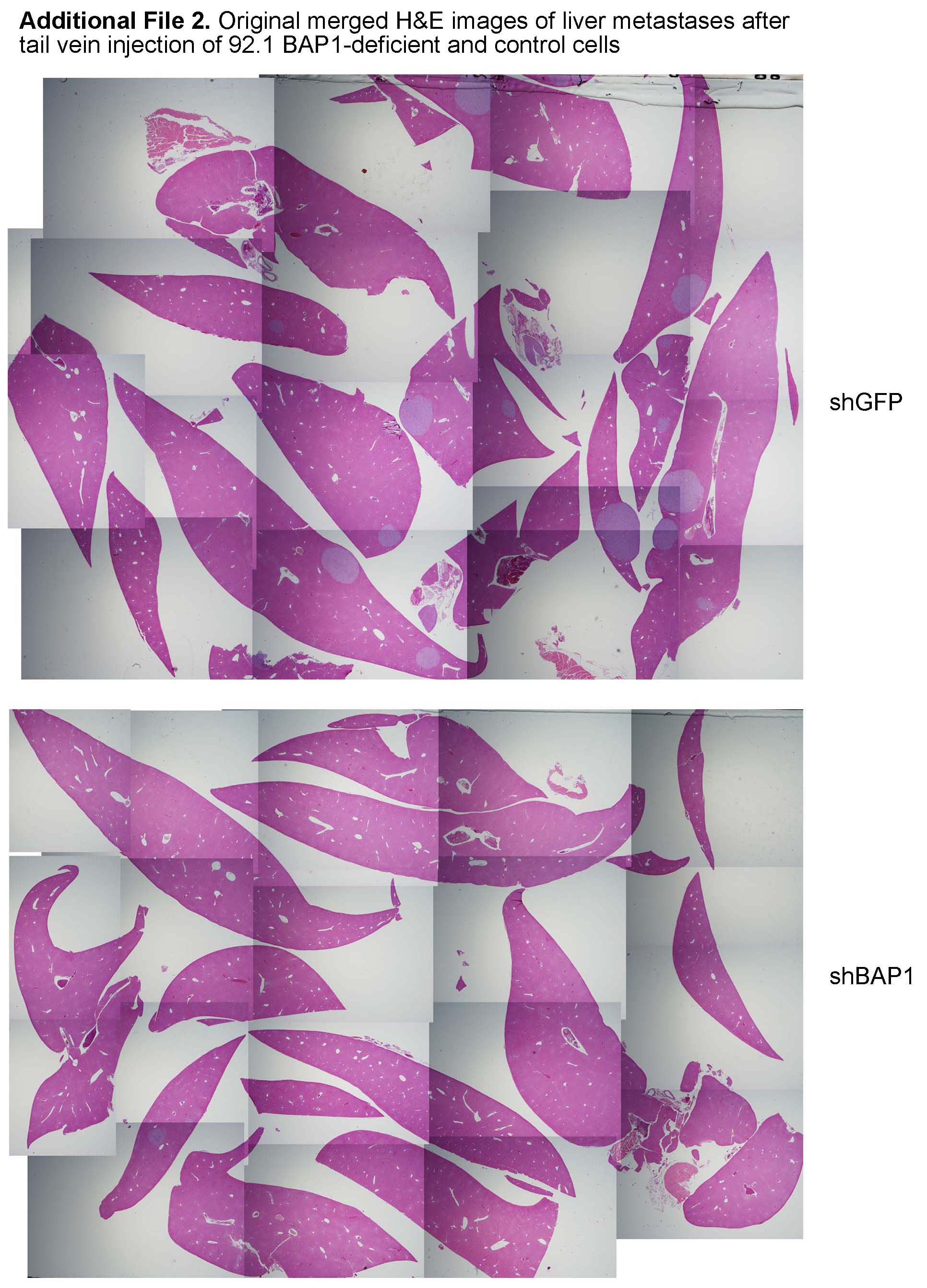

Supplement: Additional file 2 — Original H&E images of liver metastases. The original merged H&E images of liver metastases after tail vein injection of 92.1 BAP1-deficient and control cells. [file 1471-2407-13-371-S2.jpeg]

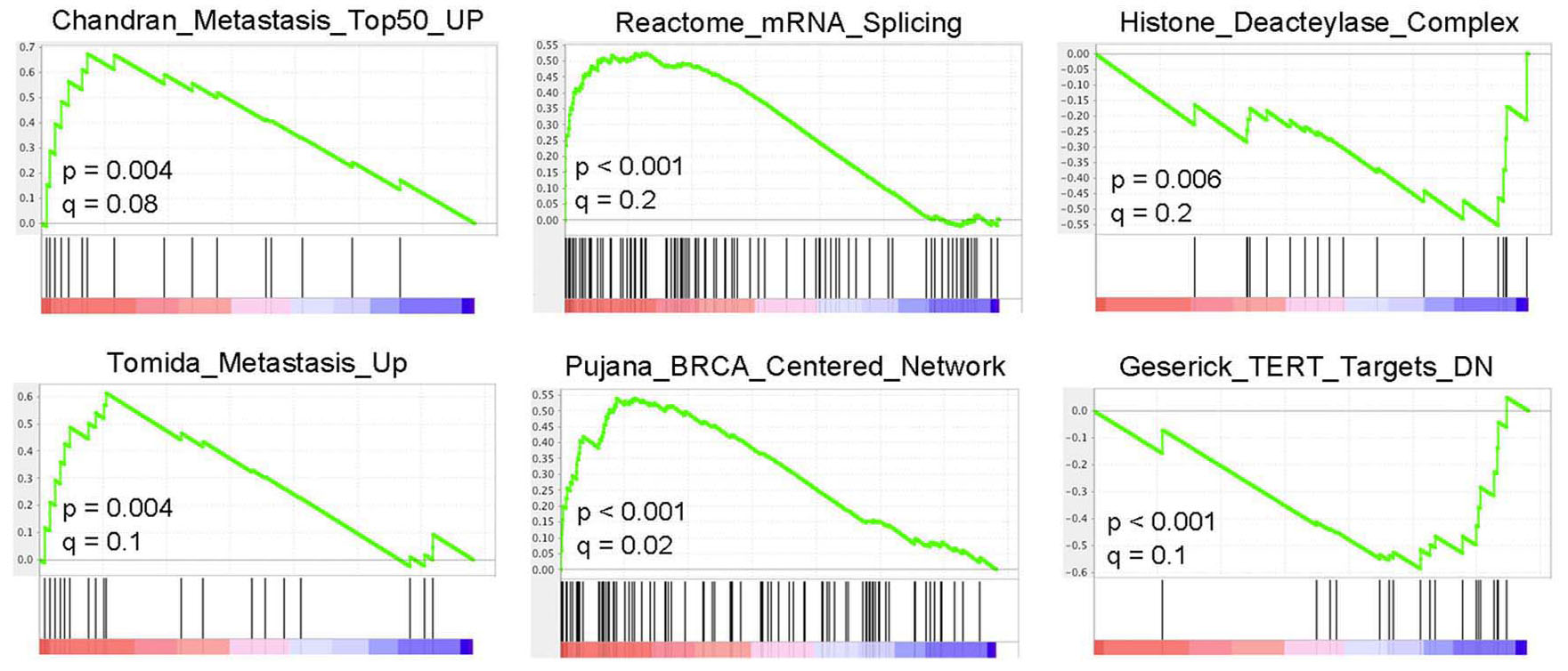

Supplement: Additional file 7 — Six of the top gene sets after GSEA analysis. Six of the top gene sets significantly enriched in BAP1-deficient cells based on GSEA analysis. [file 1471-2407-13-371-S7.jpeg]

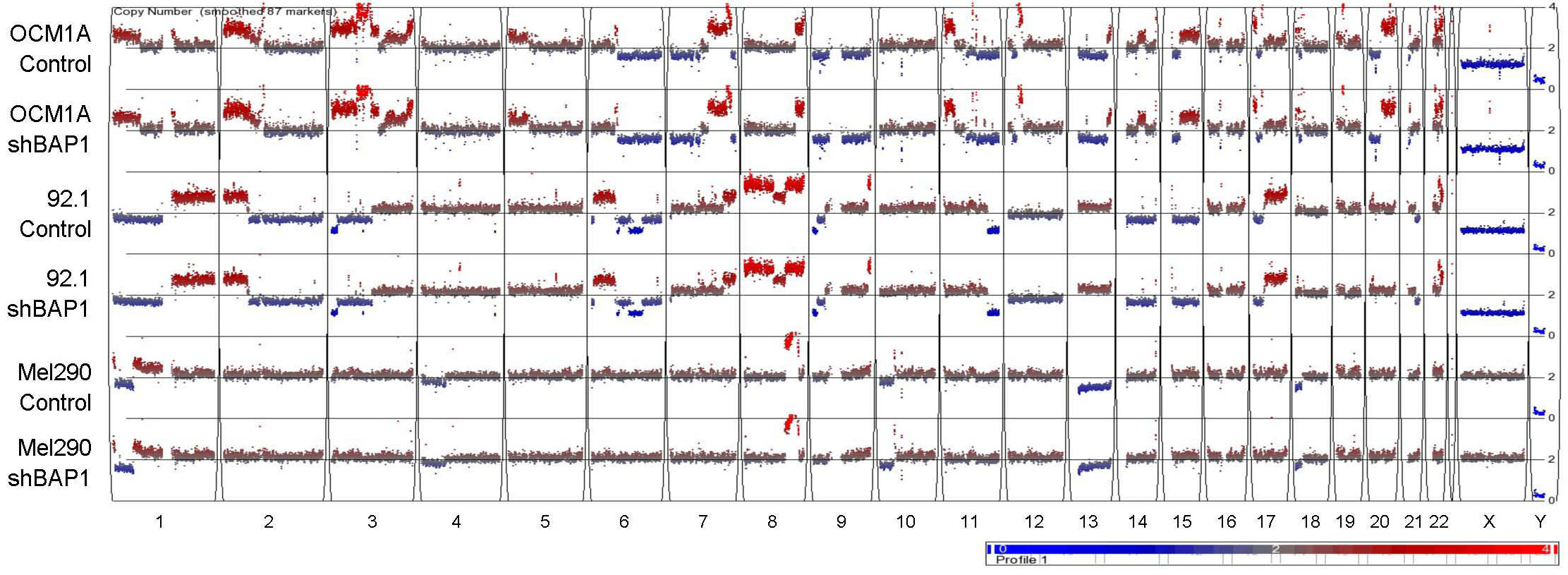

Supplement: Additional file 8 — Single nucleotide polymorphism arrays. Copy number analysis of single nucleotide polymorphism (SNP) arrays that were performed on three uveal melanoma cell lines (OCM1A, 92.1 and Mel290) expressing either BAP1 or control shRNA for four weeks. [file 1471-2407-13-371-S8.jpeg]

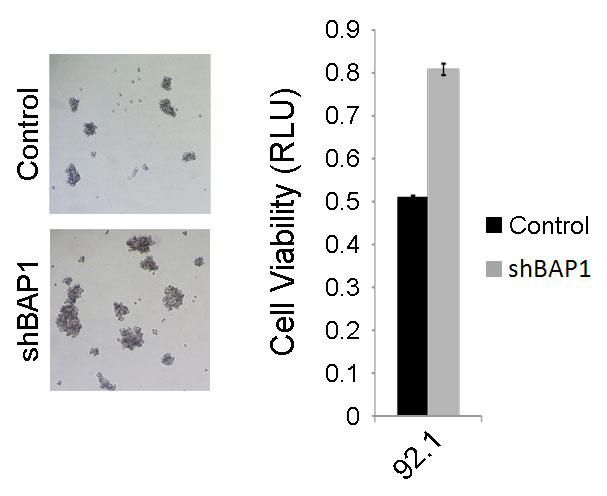

Supplement: Additional file 9 — Representative images and MTS of stable cells in stem cell conditions. (Left panels) Representative images of control and BAP1-deficient 92.1 stable cells after culture for 7 days in stem cell media in low attachment plates. (Right panel) MTS assay of control and BAP1-deficient 92.1 stable cells after culture for 7 days in stem cell media in low attachment plates. [file 1471-2407-13-371-S9.jpeg]
