# Supplementary material for: BAP1 deficiency causes loss of melanocytic cell identity in uveal melanoma
Source: BMC Cancer. 2013 Aug 5;13:371. doi: 10.1186/1471-2407-13-371 (PMC3846494; doi:10.1186/1471-2407-13-371)
Supplement: Additional file 3 — SAM analysis of gene expression profile results. Significant Analysis of Microarrays results showing genes that were up or down regulated in BAP1-deficient stable cells when compared to control cells. Only genes with a false discovery rate of less than 10% are shown. [file 1471-2407-13-371-S3.pdf]

Additional File 3. SAM analysis showing genes that were up or down regulated in BAP1-deficient stable cells when compared to control cells[1]

Direction after BAP1 knockdown	Gene symbol	Gene name
Down	CKMT1A	creatine kinase, mitochondrial 1A
Down	FNBP1L	formin binding protein 1-like
Down	GABARAPL2	GABA(A) receptor-associated protein-like 2
Down	HECTD2	HECT domain containing E3 ubiquitin protein ligase 2
Down	HNRNPH2	heterogeneous nuclear ribonucleoprotein H2 (H')
Down	TNPO1	transportin 1
Up	ANLN	anillin, actin binding protein
Up	ARPC2	actin related protein 2/3 complex, subunit 2
Up	BNIP3L	BCL2/adenovirus E1B 19kDa interacting protein 3-like
Up	BTF3	basic transcription factor 3
Up	C2orf80	chromosome 2 open reading frame 80
Up	CD68	CD68 molecule
Up	CDK14	cyclin-dependent kinase 14
Up	CNIH	cornichon homolog
Up	COMMD5	COMM domain containing 5
Up	DDX24	DEAD (Asp-Glu-Ala-Asp) box polypeptide 24
Up	DNAJC17	DnaJ (Hsp40) homolog, subfamily C, member 17
Up	ENDOG	endonuclease G
Up	ENY2	enhancer of yellow 2 homolog
Up	EXOC7	exocyst complex component 7
Up	EXTL2	exostoses (multiple)-like 2
Up	F13B	coagulation factor XIII, B polypeptide
Up	FAM175B	FAM175B family with sequence similarity 175
Up	FBXO18	F-box protein, helicase, 18
Up	FRRS1	ferric-chelate reductase 1
Up	H2AFY	H2A histone family, member Y
Up	JARID2	jumonji, AT rich interactive domain
Up	JOSD1	Josephin domain containing 1
Up	LAPTM4A	lysosomal protein transmembrane 4 alpha
Up	LMTK2	lemur tyrosine kinase 2
Up	LSM12	LSM12 homolog (S. cerevisiae)
Up	MAPK1	Mitogen-activated protein kinase 1
Up	MBNL1	muscleblind-like splicing regulator 1
Up	MGC27345	hypothetical protein MGC27345
Up	MRFAP1L1	Morf4 family associated protein 1-like 1
Up	MRPL40	mitochondrial ribosomal protein L40
Up	MRPL54	mitochondrial ribosomal protein L54
Up	MRPS17	mitochondrial ribosomal protein S17
Up	MRPS33	mitochondrial ribosomal protein S33
Up	MUC3A	mucin 3A, cell surface associated
Up	NDUFB6	NADH dehydrogenase (ubiquinone) 1 beta subcomplex, 6
Up	NPRL3	nitrogen permease regulator-like 3
Up	NR2F1	nuclear receptor subfamily 2, group F, member 1
Up	NRBP1	nuclear receptor binding protein 1
Up	OTUD4	OTU domain containing 4
Up	PDIK1L	PDLIM1 interacting kinase 1 like
Up	PPHLN1	periphilin 1
Up	PRPF4	PRP4 pre-mRNA processing factor 4 homolog (yeast)
Up	RAC1	ras-related C3 botulinum toxin substrate 1 (rho family, small GTP binding protein Rac1)
Up	RBCK1	RanBP-type and C3HC4-type zinc finger containing 1
Up	RBM28	RNA binding motif protein 28
Up	RBMY1J	RNA binding motif protein, Y-linked, family 1, member J
Up	REXO1	REX1, RNA exonuclease 1 homolog
Up	RHBDL1	rhomboid, veinlet-like 1 (Drosophila)
Up	SARNP	SAP domain containing ribonucleoprotein
Up	SCAMP3	secretory carrier membrane protein 3
Up	SEC31A	SEC31 homolog A (S. cerevisiae)
Up	SF3B5	splicing factor 3b, subunit 5, 10kDa
Up	SNORD91A	small nucleolar RNA, C/D box 91A
Up	SNRPN	small nuclear ribonucleoprotein polypeptide N
Up	UAP1	UDP-N-acteylglucosamine pyrophosphorylase 1
Up	UBE2K	UBE2K ubiquitin-conjugating enzyme E2K
Up	UBE2O	ubiquitin-conjugating enzyme E2O
Up	UBL5	ubiquitin-like 5
Up	USP21	ubiquitin specific peptidase 21
Up	WNT7B	wingless-type MMTV integration site family, member 7B
Up	ZBTB7A	Zinc finger and BTB domain containing 7A

[1]Genes with a false discovery rate under 10%
