# Supplementary material for: BAP1 deficiency causes loss of melanocytic cell identity in uveal melanoma
Source: BMC Cancer. 2013 Aug 5;13:371. doi: 10.1186/1471-2407-13-371 (PMC3846494; doi:10.1186/1471-2407-13-371)
Supplement: Additional file 4 — Validation of select genes. qPCR validation in three uveal melanoma cell lines of select genes that were significantly altered after SAM analysis of gene expression profile results. [file 1471-2407-13-371-S4.pdf]

Additional File 4. Validation of select genes

Direction after BAP1 knockdown	Gene Symbol	OCM1A Fold Change	OCM1A T-Test	92.1 Fold Change	92.1 T-Test	Mel290 Fold Change	Mel290 T-Test	Gene Name
Up	EXTL2	2.20	0.000020	1.40	0.005000	1.40	0.000500	exostoses (multiple)-like 2
Up	FAM175B	1.60	0.003000	1.50	0.005000	1.40	0.008000	FAM175B family with sequence similarity 175
Up	MBNL1	1.30	0.001000	2.10	0.000800	2.10	0.000001	muscleblind-like splicing regulator 1
Up	PRPF4	1.60	0.010000	1.50	0.000100	1.40	0.009000	PRP4 pre-mRNA processing factor 4 homolog (yeast)
Up	UBE2K	2.10	0.000002	1.30	0.003000	1.80	0.000300	UBE2K ubiquitin-conjugating enzyme E2K
Down	CKMT1A	-3.40	0.000200	-5.30	0.000600	-6.80	0.000004	creatine kinase, mitochondrial 1A
Down	HECTD2	-2.50	0.030000	-1.30	0.003000	-2.00	0.002000	HECT domain containing E3 ubiquitin protein ligase 2
Down	TNPO1	-1.80	0.000400	-1.20	0.005000	-1.40	0.030000	transportin 1
