# Supplementary material for: BAP1 deficiency causes loss of melanocytic cell identity in uveal melanoma
Source: BMC Cancer. 2013 Aug 5;13:371. doi: 10.1186/1471-2407-13-371 (PMC3846494; doi:10.1186/1471-2407-13-371)
Supplement: Additional file 5 — Top gene sets after GSEA analysis. A list of gene sets with a p < 0.005 after gene set enrichment analysis of BAP1-deficient cells. [file 1471-2407-13-371-S5.pdf]

Additional File 5. Top gene sets after GSEA analysis[1]

Direction after BAP1 knockdown	Name	Size	ES	NES	NOM p-val	FDR q-val
Up	BIOCARTA_SPRY_PATHWAY	18	0.719	1.93	0.002	0.137
Up	NIKOLSKY_BREAST_CANCER_1Q21_AMPLICON	37	0.605	1.92	0.000	0.108
Up	SONG_TARGETS_OF_IE86_CMV_PROTEIN	47	0.578	1.91	0.000	0.164
Up	LIU_COMMON_CANCER_GENES	40	0.586	1.90	0.001	0.078
Up	LY_AGING_OLD_DN	47	0.565	1.89	0.000	0.101
Up	SHEPARD_BMYB_TARGETS	60	0.535	1.89	0.000	0.062
Up	YAO_TEMPORAL_RESPONSE_TO_PROGESTERONE_CLUSTER_13	159	0.471	1.87	0.000	0.092
Up	BENPORATH_PROLIFERATION	144	0.472	1.87	0.000	0.057
Up	HU_ANGIOGENESIS_DN	30	0.616	1.86	0.000	0.048
Up	REACTOME_TELOMERE_MAINTENANCE	75	0.509	1.85	0.000	0.229
Up	CHEOK_RESPONSE_TO_HD_MTX_DN	24	0.633	1.84	0.001	0.055
Up	MORI_MATURE_B_LYMPHOCYTE_DN	57	0.531	1.84	0.001	0.050
Up	BIOCARTA_HIVNEF_PATHWAY	56	0.528	1.83	0.000	0.207
Up	CAFFAREL_RESPONSE_TO_THC_24HR_5_DN	48	0.539	1.81	0.001	0.066
Up	FERREIRA_EWINGS_SARCOMA_UNSTABLE_VS_STABLE_UP	147	0.454	1.79	0.000	0.072
Up	CHANDRAN_METASTASIS_TOP50_UP	16	0.675	1.78	0.005	0.078
Up	ODONNELL_TFRC_TARGETS_DN	132	0.453	1.76	0.000	0.096
Up	BIOCARTA_RACCYCD_PATHWAY	26	0.592	1.74	0.001	0.336
Up	REACTOME_G1_S_TRANSITION	100	0.457	1.74	0.000	0.288
Up	CAFFAREL_RESPONSE_TO_THC_DN	26	0.588	1.73	0.004	0.118
Up	TOMIDA_METASTASIS_UP	20	0.614	1.72	0.004	0.113
Up	PUJANA_BREAST_CANCER_WITH_BRCA1_MUTATED_UP	54	0.497	1.72	0.004	0.454
Up	MISSIAGLIA_REGULATED_BY_METHYLATION_DN	97	0.463	1.72	0.000	0.108
Up	NIKOLSKY_BREAST_CANCER_16P13_AMPLICON	116	0.445	1.72	0.000	0.104
Up	GARGALOVIC_RESPONSE_TO_OXIDIZED_PHOSPHOLIPIDS_TURQUOISE_DN	52	0.493	1.72	0.005	0.093
Up	REACTOME_E2F_MEDIATED_REGULATION_OF_DNA_REPLICATION	31	0.556	1.72	0.004	0.323
Up	KOBAYASHI_EGFR_SIGNALING_24HR_DN	249	0.414	1.72	0.000	0.383
Up	ROSS_AML_OF_FAB_M7_TYPE	68	0.479	1.71	0.000	0.092
Up	REACTOME_MRNA_SPLICING_MINOR_PATHWAY	42	0.520	1.71	0.004	0.295
Up	BHATTACHARYA_EMBRYONIC_STEM_CELL	75	0.465	1.70	0.000	0.362
Up	KEGG_SPLICEOSOME	126	0.441	1.70	0.000	0.260
Up	TIEN_INTESTINE_PROBIOTICS_24HR_UP	550	0.392	1.70	0.000	0.101
Up	WEI_MYCN_TARGETS_WITH_E_BOX	777	0.385	1.70	0.000	0.097
Up	ZHANG_RESPONSE_TO_CANTHARIDIN_DN	60	0.484	1.69	0.001	0.352
Up	NAKAMURA_METASTASIS_MODEL_UP	43	0.519	1.68	0.003	0.314
Up	WINNEPENNINCKX_MELANOMA_METASTASIS_UP	157	0.423	1.68	0.000	0.286
Up	FERRANDO_T_ALL_WITH_MLL_ENL_FUSION_DN	77	0.457	1.67	0.001	0.277
Up	REACTOME_RNA_POLYMERASE_I_PROMOTER_CLEARANCE	81	0.459	1.67	0.003	0.233
Up	AMUNDSON_GENOTOXIC_SIGNATURE	85	0.445	1.66	0.001	0.130
Up	ST_FAS_SIGNALING_PATHWAY	59	0.474	1.65	0.005	0.226
Up	KEGG_NUCLEOTIDE_EXCISION_REPAIR	44	0.494	1.65	0.003	0.215
Up	REACTOME_PACKAGING_OF_TELOMERE_ENDS	48	0.496	1.65	0.004	0.212
Up	ALONSO_METASTASIS_UP	156	0.414	1.64	0.000	0.291
Up	REACTOME_VIF_MEDIATED_DEGRADATION_OF_APOBEC3G	47	0.490	1.64	0.004	0.196
Up	BLUM_RESPONSE_TO_SALIRASIB_DN	335	0.384	1.63	0.000	0.250
Up	REACTOME_S_PHASE	102	0.432	1.63	0.004	0.204
Up	REACTOME_SYNTHESIS_OF_DNA	88	0.439	1.63	0.002	0.195
Up	REACTOME_PROCESSING_OF_CAPPED_INTRON_CONTAINING_PRE_MRNA	137	0.406	1.63	0.000	0.190
Up	STARK_PREFRONTAL_CORTEX_22Q11_DELETION_DN	435	0.376	1.62	0.000	0.144
Up	RHODES_CANCER_META_SIGNATURE	54	0.462	1.62	0.005	0.270
Up	PUJANA_BRCA_CENTERED_NETWORK	93	0.429	1.61	0.001	0.152
Up	GRAHAM_NORMAL_QUIESCENT_VS_NORMAL_DIVIDING_DN	87	0.433	1.61	0.005	0.148
Up	MANALO_HYPOXIA_DN	284	0.383	1.61	0.000	0.144
Up	KEGG_PYRIMIDINE_METABOLISM	97	0.431	1.61	0.004	0.207
Up	REACTOME_MRNA_SPLICING	106	0.425	1.61	0.000	0.201
Up	MARKEY_RB1_ACUTE_LOF_DN	205	0.391	1.61	0.001	0.143
Up	REACTOME_ELONGATION_AND_PROCESSING_OF_CAPPED_TRANSCRIPTS	133	0.409	1.60	0.002	0.214
Up	REACTOME_HOST_INTERACTIONS_OF_HIV_FACTORS	120	0.413	1.60	0.001	0.213
Up	ROSTY_CERVICAL_CANCER_PROLIFERATION_CLUSTER	139	0.406	1.60	0.002	0.161
Up	WONG_MITOCHONDRIA_GENE_MODULE	209	0.390	1.60	0.000	0.310
Up	PUJANA_XPRSS_INT_NETWORK	166	0.396	1.59	0.000	0.162
Up	KEGG_PURINE_METABOLISM	158	0.395	1.59	0.000	0.216
Up	REACTOME_DNA_REPLICATION_PRE_INITIATION	75	0.436	1.58	0.003	0.222
Up	REACTOME_RNA_POLYMERASE_I_III_AND_MITOCHONDRIAL_TRANSCRIPTION	119	0.404	1.57	0.002	0.217
Up	REACTOME_FORMATION_AND_MATURATION_OF_MRNA_TRANSCRIPT	151	0.392	1.57	0.000	0.214
Up	FOURNIER_ACINAR_DEVELOPMENT_LATE_2	273	0.375	1.56	0.000	0.362
Up	MUELLER_PLURINET	294	0.371	1.56	0.000	0.377
Up	LASTOWSKA_NEUROBLASTOMA_COPY_NUMBER_UP	170	0.385	1.55	0.001	0.198
Up	SOTIRIOU_BREAST_CANCER_GRADE_1_VS_3_UP	150	0.390	1.55	0.002	0.201
Up	GRADE_COLON_AND_RECTAL_CANCER_UP	221	0.371	1.55	0.000	0.389
Up	REACTOME_AXON_GUIDANCE	159	0.387	1.55	0.003	0.208
Up	KRIGE_RESPONSE_TO_TOSEDOSTAT_6HR_DN	872	0.349	1.54	0.000	0.389
Up	REACTOME_INFLUENZA_VIRAL_RNA_TRANSCRIPTION_AND_REPLICATION	100	0.406	1.53	0.004	0.212
Up	GRAHAM_CML_DIVIDING_VS_NORMAL_QUIESCENT_UP	182	0.379	1.53	0.001	0.219
Up	REACTOME_CELL_CYCLE_MITOTIC	301	0.360	1.53	0.000	0.205
Up	SPIELMAN_LYMPHOBLAST_EUROPEAN_VS_ASIAN_UP	469	0.351	1.52	0.000	0.222
Up	YAO_TEMPORAL_RESPONSE_TO_PROGESTERONE_CLUSTER_17	167	0.377	1.52	0.001	0.422
Up	DEBIASI_APOPTOSIS_BY_REOVIRUS_INFECTION_DN	223	0.369	1.52	0.001	0.412
Up	REACTOME_TRANSCRIPTION	195	0.370	1.51	0.001	0.214

Up	KAUFFMANN_DNA_REPAIR_GENES	204	0.369	1.51	0.003	0.228
Up	WANG_CLIM2_TARGETS_DN	172	0.371	1.50	0.003	0.226
Up	KRIGE_RESPONSE_TO_TOSEDOSTAT_24HR_DN	967	0.338	1.50	0.000	0.412
Up	GRADE_COLON_CANCER_UP	712	0.341	1.50	0.000	0.412
Up	LIU_SOX4_TARGETS_DN	303	0.354	1.50	0.000	0.234
Up	GOLDRATH_ANTIGEN_RESPONSE	315	0.349	1.49	0.001	0.405
Up	BUYTAERT_PHOTODYNAMIC_THERAPY_STRESS_DN	624	0.339	1.49	0.000	0.244
Up	BENPORATH_MYC_MAX_TARGETS	762	0.337	1.48	0.000	0.247
Up	PUJANA_BRCA2_PCC_NETWORK	416	0.342	1.47	0.001	0.253
Up	BERENJENO_TRANSFORMED_BY_RHOA_UP	489	0.336	1.46	0.000	0.262
Up	BASAKI_YBX1_TARGETS_UP	287	0.346	1.46	0.000	0.254
Up	NAKAMURA_TUMOR_ZONE_PERIPHERAL_VS_CENTRAL_UP	276	0.346	1.45	0.003	0.265
Up	NUYTTEN_EZH2_TARGETS_DN	914	0.327	1.44	0.000	0.264
Up	REACTOME_GENE_EXPRESSION	423	0.331	1.43	0.000	0.276
Up	PUJANA_CHEK2_PCC_NETWORK	758	0.320	1.42	0.000	0.292
Up	BENPORATH_ES_1	372	0.331	1.41	0.002	0.302
Up	GARY_CD5_TARGETS_DN	422	0.329	1.41	0.000	0.299
Up	NIKOLSKY_BREAST_CANCER_17Q21_Q25_AMPLICON	330	0.335	1.41	0.001	0.300
Up	BORCZUK_MALIGNANT_MESOTHELIOMA_UP	298	0.336	1.41	0.002	0.300
Up	MOOTHA_HUMAN_MITODB_6_2002	421	0.326	1.41	0.002	0.460
Up	HORIUCHI_WTAP_TARGETS_DN	299	0.333	1.40	0.002	0.312
Up	BENPORATH_CYCLING_GENES	638	0.319	1.40	0.000	0.310
Up	GINESTIER_BREAST_CANCER_ZNF217_AMPLIFIED_DN	316	0.327	1.39	0.004	0.311
Up	MULLIGHAN_MLL_SIGNATURE_1_UP	372	0.324	1.39	0.002	0.309
Up	BLALOCK_ALZHEIMERS_DISEASE_INCIPIENT_UP	383	0.323	1.38	0.003	0.471
Up	LINDGREN_BLADDER_CANCER_CLUSTER_1_DN	369	0.319	1.37	0.003	0.316
Up	ZHOU_INFLAMMATORY_RESPONSE_LIVE_DN	365	0.318	1.36	0.003	0.315
Up	MILI_PSEUDOPODIA_HAPTOTAXIS_UP	457	0.315	1.36	0.003	0.468
Up	ZHOU_INFLAMMATORY_RESPONSE_LPS_DN	365	0.318	1.36	0.005	0.314
Up	SHEDDEN_LUNG_CANCER_POOR_SURVIVAL_A6	451	0.315	1.35	0.002	0.473
Up	KIM_WT1_TARGETS_DN	448	0.311	1.34	0.001	0.328
Up	SHEN_SMARCA2_TARGETS_UP	414	0.309	1.33	0.005	0.338
Up	RODRIGUES_THYROID_CARCINOMA_POORLY_DIFFERENTIATED_UP	611	0.301	1.32	0.005	0.350
Up	ACEVEDO_LIVER_TUMOR_VS_NORMAL_ADJACENT_TISSUE_UP	852	0.299	1.32	0.000	0.524
Up	MILI_PSEUDOPODIA_HAPTOTAXIS_DN	606	0.295	1.30	0.002	0.529
Up	BENPORATH_NANOG_TARGETS	962	0.278	1.23	0.005	0.468
Down	MASSARWEH_TAMOXIFEN_RESISTANCE_UP	555	-0.202	-1.12	0.000	0.595
Down	GOZGIT_ESR1_TARGETS_DN	731	-0.201	-1.14	0.000	0.529
Down	STEIN_ESRRA_TARGETS	518	-0.207	-1.15	0.000	0.561
Down	SATO_SILENCED_BY_METHYLATION_IN_PANCREATIC_CANCER_1	408	-0.212	-1.16	0.000	0.565
Down	KRIGE_RESPONSE_TO_TOSEDOSTAT_24HR_UP	739	-0.209	-1.16	0.000	0.570
Down	NUYTTEN_EZH2_TARGETS_UP	973	-0.206	-1.16	0.000	0.551
Down	ENK_UV_RESPONSE_EPIDERMIS_DN	505	-0.216	-1.22	0.000	0.518
Down	CREIGHTON_ENDOCRINE_THERAPY_RESISTANCE_3	693	-0.213	-1.23	0.000	0.521
Down	ONDER_CDH1_TARGETS_2_DN	456	-0.230	-1.27	0.000	0.463
Down	MCBRYAN_PUBERTAL_BREAST_6_7WK_UP	176	-0.262	-1.30	0.000	0.461
Down	ACEVEDO_LIVER_CANCER_DN	529	-0.243	-1.33	0.000	0.437
Down	GARY_CD5_TARGETS_UP	460	-0.239	-1.34	0.000	0.428
Down	KAAB_HEART_ATRIUM_VS_VENTRICLE_DN	260	-0.254	-1.35	0.000	0.417
Down	BLUM_RESPONSE_TO_SALIRASIB_UP	244	-0.269	-1.36	0.000	0.413
Down	VANTVEER_BREAST_CANCER_ESR1_UP	139	-0.298	-1.44	0.000	0.411
Down	IWANAGA_CARCINOGENESIS_BY_KRAS_UP	147	-0.295	-1.44	0.000	0.448
Down	BROWNE_HCMV_INFECTION_18HR_UP	175	-0.288	-1.45	0.000	0.458
Down	KEGG_CELL_ADHESION_MOLECULES_CAMS	134	-0.298	-1.46	0.000	0.428
Down	BHATI_G2M_ARREST_BY_2METHOXYESTRADIOL_DN	124	-0.312	-1.46	0.000	0.475
Down	SEKI_INFLAMMATORY_RESPONSE_LPS_UP	74	-0.339	-1.48	0.004	0.508
Down	AMIT_EGF_RESPONSE_120_HELA	65	-0.358	-1.52	0.000	0.326
Down	KIM_WT1_TARGETS_12HR_UP	156	-0.306	-1.53	0.000	0.333
Down	KEGG_GLYCOLYSIS_GLUCONEOGENESIS	62	-0.368	-1.54	0.005	0.368
Down	MAHAJAN_RESPONSE_TO_IL1A_DN	62	-0.366	-1.57	0.005	0.426
Down	REACTOME_TRNA_AMINOACYLATION	39	-0.421	-1.59	0.004	0.300
Down	ROSS_ACUTE_MYELOID_LEUKEMIA_CBF	81	-0.379	-1.64	0.000	0.191
Down	SAGIV_CD24_TARGETS_DN	45	-0.425	-1.64	0.004	0.362
Down	RORIE_TARGETS_OF_EWSR1_FLI1_FUSION_UP	25	-0.480	-1.69	0.000	0.340
Down	REACTOME_METABOLISM_OF_AMINO_ACIDS	162	-0.353	-1.73	0.000	0.188
Down	BASSO_CD40_SIGNALING_UP	101	-0.374	-1.73	0.000	0.314
Down	GRABARCZYK_BCL11B_TARGETS_DN	54	-0.437	-1.77	0.000	0.151
Down	YAO_TEMPORAL_RESPONSE_TO_PROGESTERONE_CLUSTER_6	65	-0.427	-1.79	0.000	0.247
Down	REACTOME_CYTOSOLIC_TRNA_AMINOACYLATION	22	-0.558	-1.84	0.000	0.120
Down	GESERICK_TERT_TARGETS_DN	19	-0.587	-1.85	0.000	0.104
Down	HEIDENBLAD_AMPLIFIED_IN_PANCREATIC_CANCER	31	-0.516	-1.87	0.000	0.114
Down	KEGG_ARGININE_AND_PROLINE_METABOLISM	54	-0.461	-1.87	0.000	0.119
Down	TIEN_INTESTINE_PROBIOTICS_24HR_DN	206	-0.369	-1.92	0.000	0.100
Down	HOOI_ST7_TARGETS_UP	87	-0.431	-1.92	0.000	0.147
Down	WEBER_METHYLATED_HCP_IN_FIBROBLAST_DN	30	-0.533	-1.92	0.003	0.140
Down	KRIGE_AMINO_ACID_DEPRIVATION	26	-0.583	-2.03	0.000	0.106
Down	MAHADEVAN_RESPONSE_TO_MP470_DN	16	-0.691	-2.08	0.003	0.049
Down	REACTOME_BRANCHED_CHAIN_AMINO_ACID_CATABOLISM	17	-0.717	-2.15	0.000	0.005
Down	KEGG_VALINE_LEUCINE_AND_ISOLEUCINE_DEGRADATION	44	-0.564	-2.17	0.000	0.009

[1]Gene sets with a nominal P-value≤0.005
