# Supplementary material for: BAP1 deficiency causes loss of melanocytic cell identity in uveal melanoma
Source: BMC Cancer. 2013 Aug 5;13:371. doi: 10.1186/1471-2407-13-371 (PMC3846494; doi:10.1186/1471-2407-13-371)
Supplement: Additional file 6 — Enriched genes associated with each gene set category. A list of genes enriched in at least two gene sets within a given category after GSEA analysis of BAP1-deficient stable cells when compared to control cells. The categories listed are those referred to in Figure 5c. [file 1471-2407-13-371-S6.pdf]

Additional File 6. Enriched genes associated with each gene set category[1]

Cell Cycle/ Proliferation	Development/ Stem Cell	RNA Metabolism/ Splicing	DNA Damage/ Telomere Maintenance	Metastasis	Epigenetic Regulation	Amino Acid Metabolism	BRCA1/2 Pathway	Mitochondria
ANAPC11	ABHD11	CCAR1	ATR	AK3L1	ATP6V0E1	ACAT1	ASF1A	ABCB6
ANLN	ACTR1A	CD2BP2	BRCA1	ANLN	CAPZA2	ADC	BAT1	AK2
ASPM	ADD3	CSTF1	CHAF1A	CCT5	CHMP5	AGMAT	CDKN2C	ATP5E
BIRC5	ARL6IP6	CSTF3	CHEK2	CKS1B	CSE1L	ALDH1B1	CHEK2	ATP5I
BRCA1	ASPM	CTDP1	DNA2	DAD1	SERINC3	ALDH2	DNA2	ATP5J
BUB1	ATP11C	ERCC2	ERCC1	GRB2		ALDH6A1	DNMT1	ATP5J2
CASP8AP2	ATP1B1	GTF2H1	ERCC2	H2AFV		ARG2	EXOSC8	ATP5L
CCDC99	ATP2C1	HNRNPA0	ERCC5	HTRA2		ASNS	EZH2	GPX4
CCNB1	BAT1	HNRNPA1	ERCC6	KIF11		AUH	HMMR	HMGCS2
CCNF	BCLAF1	HNRNPA2B1	EXO1	MRPS22		BCAT1	ILF3	MRPL13
CD36	BIRC5	HNRNPR	FEN1			BCAT2	KIFC1	MRPS15
CDC25A	BRIX1	HNRNPU	GTF2H1			CKMT1A	LBR	MRPS18C
CDCA3	BUB1	HNRNPUL1	GTF2H5			CKMT1B	MCM5	MRPS33
CDKN3	C14ORF106	LSM2	H2AFX			GATM	MTF2	NDUFA3
CDT1	C14ORF115	NCBP2	HIST1H2AC			GLUD1	PRPF4	NDUFA8
CENPF	C19ORF42	NFX1	HIST1H2AE			GOT1	RAD51C	NDUFB1
CKAP2	CACHD1	NHP2L1	HIST1H2BD			GOT2	RAD54L	NDUFB3
CKS1B	CASP9	PABPN1	HIST1H2BG			HIBADH	RBBP4	NDUFB6
CKS2	CCNB1	PAPOLA	HIST1H2BJ			IVD	RFC4	NDUFC1
CNIH4	CDC25A	PCBP2	HIST1H2BK			MCCC2	RPA1	NDUFS5
CPA3	CDC42EP3	PHF5A	HIST1H2BO			OAT	RPIA	NDUFS8
CSE1L	CDCA3	POLR2A	HIST1H4E			ODC1	RQCD1	NDUFV2
CUL1	CDKN3	POLR2F	HIST1H4F			SAT1	RRM1	NFS1
DNA2	CDT1	POLR2H	HIST1H4H			SRM	SKP2	PCCB
E2F3	CETN3	POLR2I	HIST1H4J				SNRPA1	TIMM23
ECT2	CHEK2	POLR2L	HIST1H4K				SNRPB	TSPO
ERCC6L	CKS1B	PRPF4	HIST2H2AA3				TFDP1	VDAC3
EXO1	CKS2	SF3A2	HIST2H2AC				TMPO	
EZH2	CSE1L	SF3B14	HIST2H2BE				TOPBP1	
FABP5	CSTF3	SF3B5	MSH6				UBE2C	
FBXO5	CYP26A1	SFRS3	POLA1				UNG	
FEN1	DEPDC1	SFRS7	POLA2				USP1	
FZR1	ECT2	SNRNP70	POLE4				XPO1	
GINS3	ERBB2	SNRPA1	POT1					
GPSM2	EXOSC5	SNRPB	RAD51C					
GTSE1	FABP5	SNRPB2	RAD54L					
H2AFV	FAM64A	SNRPD1	RBBP4					
H2AFX	FBL	SNRPF	RFC4					
HELLS	FBXL14	SNRPG	RPA1					
HMMR	FEN1	TCEB1	RPA2					
HN1	FKBP5	TCEB2	RPA3					
HSPA14	FLNA	TCEB3	RRM1					
KIAA0101	FXR1	THOC4	RUVBL1					
KIF11	FZD7	U2AF1	SHFM1					
KIF14	GAL	U2AF2	SUMO1					
KIFC1	GDF3		TERF1					
LBR	GEMIN6		TERT					
LEPR	GMPR2		TINF2					
LRP8	H2AFV		TOPBP1					
MCM5	HELLS		UBE2I					
MCM7	HMGA1		UNG					
MDM2	HMMR		XPA					
MELK	IGF2BP3							
MINPP1	ILF3							
MLF1IP	INF2							
MRPS15	JARID2							
NANOG	KCTD2							
NCAPG	KIAA0101							
NUF2	KIF14							
NUP85	KNTC1							
NUSAP1	KRT8							
OIP5	LARP1							
ORC1L	LBR							
ORC4L	LDHA							
ORC5L	LRP8							
PARP1	LRRN1							
PDSS1	LXN							
PKMYT1	MAP7							
POLA1	MBNL1							
POLA2	MELK							
POLQ	MGST1							
PRC1	MKRN1							
PRG2	MSH6							
PSMA1	NANOG							
PSMA3	NEBL							
PSMA4	NIF3L1							
PSMA6	NOP16							
PSMB1	NUDT15							
PSMB10	NUF2							
PSMB3	NUSAP1							
PSMB6	OIP5							
PSMB7	ORC1L							
PSMC1	OVOL2							
PSMC2	PAPOLA							
PSMC3	PGK1							

**Cell Cycle/ Proliferation**	**Development/ Stem Cell**
PSMD11	PHGDH
PSMD12	PIH1D1
PSMD3	PITX2
PSMD6	PLAT
PSME2	POLA1
RAD51	PPAP2A
RAD54L	PRC1
RANBP1	PRDX3
RANGAP1	PREB
RFC4	PRKX
RPA1	PUS7
RPA2	RFC4
RPA3	RHOQ
RRM1	RNF138
SAC3D1	RPA3
SHCBP1	RPL13
SKP2	SCGB3A2
SMC2	SEC22C
SNRPD1	SERBP1
SPC25	SERPINE1
STAG1	SFRS7
STIL	SIN3B
SYNCRIP	SKP2
TFDP1	SLC25A11
TK1	SLC44A1
TMPO	SMARCA4
TMSB10	SMS
TOPBP1	SNRPB
UBB	SNRPF
UBE2C	SNRPN
WEE1	SPC24
XPO1	SPC25
ZNF593	SPG7
	SPRY4
	STIL
	SYPL1
	TARBP2
	TDGF1
	TEAD4
	TERF1
	TFB2M
	TK1
	TMPO
	TTLL12
	TUBG1
	UBE2C
	UNG
	USP44
	WDHD1
	WEE1
	YWHAH
	ZNF395

[1]Genes are listed only if they are enriched in at least two gene sets within a given categroy
